# A single-cell RNA sequencing atlas of circulating leukocytes from healthy and osteosarcoma affected dogs

**DOI:** 10.3389/fimmu.2023.1162700

**Published:** 2023-05-19

**Authors:** Dylan T. Ammons, R. Adam Harris, Leone S. Hopkins, Jade Kurihara, Kristen Weishaar, Steven Dow

**Affiliations:** ^1^ Department of Microbiology, Immunology and Pathology, Colorado State University, Fort Collins, CO, United States; ^2^ Flint Animal Cancer Center, Department of Clinical Sciences, Colorado State University, Fort Collins, CO, United States

**Keywords:** single cell RNA seq, PBMC (peripheral blood mononuclear cells), canine (dog), osteosarcoma, transcriptomics, cancer, immunology, MDSC (myeloid-derived suppressor cell)

## Abstract

Translationally relevant animal models are essential for the successful translation of basic science findings into clinical medicine. While rodent models are widely accessible, there are numerous limitations that prevent the extrapolation of findings to human medicine. One approach to overcome these limitations is to use animal models that are genetically diverse and naturally develop disease. For example, pet dogs spontaneously develop diseases that recapitulate the natural progression seen in humans and live in similar environments alongside humans. Thus, dogs represent a useful animal model for many areas of research. Despite the value of the canine model, species specific reagent limitations have hampered in depth characterization of canine immune cells, which constrains the conclusions that can be drawn from canine immunotherapy studies. To address this need, we used single-cell RNA sequencing to characterize the heterogeneity of circulating leukocytes in healthy dogs (n = 7) and osteosarcoma (OS) affected dogs (n = 10). We present a cellular atlas of leukocytes in healthy dogs, then employ the dataset to investigate the impact of primary OS tumors on the transcriptome of circulating leukocytes. We identified 36 unique cell populations amongst dog circulating leukocytes, with a remarkable amount of heterogeneity in CD4 T cell subtypes. In our comparison of healthy dogs and dogs with OS, we identified relative increases in the abundances of polymorphonuclear (PMN-) and monocytic (M-) myeloid-derived suppressor cells (MDSCs), as well as aberrations in gene expression within myeloid cells. Overall, this study provides a detailed atlas of canine leukocytes and investigates how the presence of osteosarcoma alters the transcriptional profiles of circulating immune cells.

## Introduction

Traditional animal models, such as rodents, have been used for decades as a steppingstone in understanding human disease. Unfortunately, due to environmental and genetic disparities between humans and model species, research findings frequently fail to translate to human medicine (1). While these animal models are valuable in preclinical research, there has been a push to incorporate the use of more biologically relevant models (2). Client owned dogs offer an excellent model to investigate novel therapeutics as they are outbred, spontaneously develop disease, and share living space with humans. Interestingly, certain cancers, such as osteosarcoma (OS), follow similar disease progression and occur at a rate roughly 75 times that of humans (3). Therefore, clinical trials in dogs allow for thorough investigation of novel therapeutics for the treatment of diseases considered to be rare and difficult to study in humans. With the increasing use of canine models to investigate novel therapeutics, there is a need to better describe canine immune cell populations.

Previous characterizations of canine immune cells have relied on antibody-based assays, such as flow cytometry, cell sorting, and immunohistochemistry, to describe cell populations (4–6). While these approaches have been fundamental in understanding canine immunology, the limited availability of markers to define cell populations has the tendency to introduce pre-selection bias. An alternative and novel approach to describe the heterogeneity of canine immune cells is to use single-cell RNA sequencing which enables the unsupervised characterization of individual immune cell transcriptomes. The flexibility of this platform allows researchers to overcome species-specific reagent barriers that have limited previous characterizations of canine immune cells.

Circulating immune cells play a key role in responding to disease and have been reported to be altered in cancer patients, with increased immune suppression being a predominate finding (7). For example, two recently defined cell populations, polymorphonuclear (PMN-) and monocytic (M-) myeloid-derived suppressor cells (MDSCs), have been reported to be expanded in individuals with cancer, and increases in circulating MDSCs have been demonstrated to have prognostic implications (8). These two distinct MDSC populations exhibit immature phenotypes with potent immune suppressive activity. Recently, canine MDSCs were characterized using flow cytometry-based assays and were defined to be MHCII negative monocytes and neutrophils isolated through density centrifugation (9, 10). Given the limited antibody selection for flow cytometry-based approaches there is a need to evaluate heterogeneity within MDSC populations, as well as investigate how all leukocyte transcriptomes are altered in dogs with cancer.

In the present study, we first complete single-cell transcriptomic analysis on seven middle-aged healthy dogs of varying breeds and genders to develop a comprehensive reference database for canine leukocytes. Then we apply the database to investigate how leukocyte abundances and transcriptomes are altered in ten dogs with spontaneously arising osteosarcoma. In depth analysis revealed key changes in immune cell abundances as well as OS induced transcriptomic changes. The data presented here highlight the differences in circulating leukocytes between healthy and osteosarcoma affected dogs, as well as provide an open-access annotated canine leukocyte database for future investigations.

## Methods

### Patient selection

Clinically healthy client-owned dogs without preexisting conditions were selected for inclusion in the healthy reference database. All dogs were followed for at least two months to ensure no disease was observed. Osteosarcoma patients were selected based on the presence of a primary tumor and all dogs were naïve to treatment. All dogs presented with radiographic evidence of OS and underwent amputation in the weeks following blood collection. All studies were approved by the Colorado State University (CSU) Institutional Animal Care and Use Committee and the CSU Clinical Review Board. All dog owners provided informed consent prior to blood donation.

### Sample preparation

Approximately 10 mL of whole blood was obtained in an EDTA collection tube then processed within 30 minutes using Ficoll Paque (Cytiva; Marlborough, MA) to complete density gradient centrifugation. Whole blood was mixed with 12 mL of phosphate buffered saline, pH = 7.40 (PBS), then layered onto 12 mL of Ficoll Paque. To isolate leukocytes, the layered sample was then centrifugated at room temperature for 40 minutes at 500 rcf with acceleration at maximum and brake off. Leukocytes were isolated through collection of the cell interface then washed one time with PBS, resuspended in 10 mL of Ammonium-Chloride-Potassium (ACK) lysis buffer for 3-7 minutes at room temperature, then washed an additional time with PBS. A final wash at 100 rcf x 15 minutes was completed to remove platelets and other small contaminates. Finally, cells were resuspended in 0.04% molecular grade BSA (Sigma-Aldrich; St. Louis, MO) in PBS and adjusted to obtain a cell count between 700-1200 cells/µL. Once in solution, leukocytes were transported to a chromium instrument (10x Genomics; Pleasanton, CA) and captured within 2 hours of preparation.

### Library preparation and sequencing

Single cells were isolated and tagged with unique cell barcodes using a Chromium controller or Chromium iX instrument (10x Genomics) with a target of 5,000 cells per sample. Single cells were isolated and processed using a Chromium Next GEM Single Cell 3' Kit v3.1 following manufacture recommended protocols. Once cells were barcoded and unique molecular identifiers (UMIs) added, a standard Illumina library preparation was completed using a dual index library construction kit (10x Genomics). Sample quality was analyzed using a TapeStation bioanalyzer and/or LabChip (Agilent Technologies; Santa Clara, CA/PerkinElmer; Waltham, MA) then submitted for sequencing on an Illumina NovaSeq 6000 sequencer (Novogene Corporation; Sacramento, CA) with a target of 50,000 150 bp paired-end reads per cell. Raw data was demultiplexed by the sequencing core then transferred for downstream analysis.

### Read mapping and quantification

A Cell Ranger analysis pipeline (version 7.0.0, 10x Genomics) was utilized to process raw FASTQ sequencing data, align reads to the canine genome, and generate a count matrix. First, the CanFam3.1 ensembl annotation (gtf) file was filtered for protein_coding, lincRNA, antisense, and immunoglobulin gene biotypes. Then a canine reference package using the filtered gtf file and CanFam3.1 genome (FASTA) was created using the cellranger mkref command. The reference package and raw FASTQ filles were then used to complete read mapping and quantification of UMIs using the cellranger count command. Each sample was aligned once using include-introns mode set to true and once with include-introns mode set to false. We observed that include-introns mode increased the sensitivity of the assay, but also increase the abundance of low-quality cell clusters. By using both methods we were then better able to filter out artifactual clusters while retaining clusters with low transcript abundance. Due to incomplete annotation of the canine genome, we also aligned the healthy dog samples to an alternate canine genome (ROS_Cfam_1.0) using Cell Ranger version 7.0.0 with include-introns mode set to true. The output count matrices obtained under each alignment protocol in the format of cell barcode x feature (column x row) were then exported and used for downstream analysis.

### Data filtering and integration

For each sample, the count matrix was imported into R using the Read10X() function then converted to a Seurat object using the CreateSeuratObject() function (11). To estimate the number of dead/poor quality cells, the percent mitochondrial reads per cell was calculated using PercentageFeatureSet() to count all reads mapped to features with the prefix “MT-”. Each object was then filtered to only retain cells which met the following requirements: 200 < nFeature_RNA < 4500, percent.mt < 10, and 500 < nCount_RNA < 20000. An initial low resolution unsupervised clustering was completed to remove contaminating red blood cells and platelets. Next, DoubletFinder was used to identify and remove putative cell doublets (12). After filtering each sample individually, all samples were integrated into one object using a SCTransform workflow (13). During this step, we regressed out the percent mitochondrial reads and percent platelet-associated reads to minimize the impacts on clustering and used 2000 features as integration anchors. Following data integration, ideal clustering parameters of each subset of data were determined using the R package clustree (14). Dimension reduction and visualization was then completed, and the data were projected using 2-dimensional, non-linear uniform manifold approximation and projection (UMAP) plots.

### Cell classification

Cells were classified using the integrated dataset containing 7 healthy and 10 OS dogs. Annotations were then transferred to the healthy only dataset to create gene lists for each cell population. Major cell population identities were assigned using compiled data gathered from singleR reference mapping, Seurat reference mapping, gene set enrichment analysis, and manual annotation based on the literature (15, 16). Further high-resolution cell identification was completed through use of independent re-clustering on cells within each major immune cell population. Confirmation of T cell subsets was completed using the AddModuleScore() function and gene lists provided in a comprehensive human T cell dataset (17). The gene lists presented as **Supplemental Data** were generated using the FindAllMarkers() function (Wilcoxon Rank Sum test) on final cell type classification in healthy only (**Supplemental Data 1**) and combined datasets (**Supplemental Data 2**). The final short gene lists were generated using a selection of the top features that define a cell type (as defined in the healthy only dataset) with preference given to unique features in the top 50 genes (weighted by adjusted P value) identified to define each cell population.

### Feature visualization

Feature expression was visualized using dot plots, feature plots, and violin plots. Selected features were chosen based on prior biological knowledge and features identified using the FindAllMarkers() function. Y-axis scales for violin plots within a figure are fixed, so they are all on the same scale. Feature plots show normalized expression for each feature and are on variable scales. Dot plots used scaled expression data which depicts deviation from the average value for a gene across the cells being sampled. Visualization of T Cell Receptor Delta Constant (TRDC) was completed by transferring normalized expression counts from the healthy samples aligned to ROS_Cfam_1.0 to the CanFam3.1 aligned healthy samples.

### Cell abundance analysis

All cell abundance comparisons were made using percentage of parent. When visualizing cellular contribution on a UMAP, all samples were down sampled to the value equal to the sample which contributed the fewest number of cells. By doing this, we obtained equal number of cells from each sample and avoided visual biasing of data presentation. To make statistical inferences on changes in cell abundances, two-sided Wilcoxon Rank Sum tests were used and exact P values were reported (18). P values less than 0.05 were considered to be statistically significant.

### Pseudotime analysis

To complete pseudotime analysis and predict cell lineages, we used Slingshot (19). Only high-quality cell clusters were used for this analysis. A starting node was selected based on biological knowledge, then an unsupervised approach was used to infer branches between clusters. Following branch identification, the Slingshot getLineages() function was used to identify predicted cell lineage pathways. Custom functions were then used to extract the data and plot the branch patterns/lineages.

### Differential gene expression analysis

Differential gene expression analysis was completed using two different approaches (1) Wilcoxon Rank Sum test (FindMarkers function) and (2) pseudobulk conversion. When possible, we used pseudobulk conversion followed by a DESeq2 pipeline to evaluate differential gene expression (20). We required a minimum of 25 cells in a sample to be included in pseudobulk conversion and only applied this approach to compare cells within clusters or between groups of cells that had limited heterogeneity. Prior to running DESeq2, low abundance features, defined as features with less than 10 raw counts across all cells sampled, were filtered out. Features that had an adjusted P value of less than 0.01 and a log2(fold change) greater than 0.58 were considered to be statistically significant. For differential gene expression analysis completed using FindMarkers(), we obtained the average normalized count of each feature grouped by classification (Y verses X), then plotted the values on a scatter plot. Values that fell below a line of y-intercept = 0; slope = 1 and were determined to be higher in the X category using the FindMarkers() function (log2(fold change) < -0.58 and adjusted P value < 0.01), were discussed as increased in X or decreased in Y. Alternatively, values that fell above a line of y-intercept = 0; slope = 1 and were determined to be higher in the Y category using the FindMarkers() function (log2(fold change) > 0.58 and adjusted P value < 0.01), were discussed as increased in Y or decreased in X. Any subsequent pathway analysis was completed using lists of upregulated or downregulated genes and the enricher() function from clusterProfiler was used with the hallmarks gene sets (21, 22).

### Human-canine homology analysis

An annotated human leukocyte dataset (blish_covid.seu.rds) consisting of 6 healthy adults was obtained from https://zenodo.org/record/4021967/ and integrated with the 7 healthy dog samples generated in this study (18). SCT normalization and integration of the merged canine and human datasets was completed using 2000 variable features, while also regressing the percentage of mitochondrial reads. Following integration, the canine and human cell type annotations were prepended with “can_” or “hu_” then SCT normalized data was used to complete hierarchical clustering of the prepended cell types, an approach adopted from Cheng et al (23). Hierarchical clustering was completed using the hclust() function with using complete Euclidean distance to complete the analysis.

### Flow cytometric analysis

A subset of cells obtained from the samples used for single-cell RNA sequencing were used for paired flow cytometric analysis of immune cell types. Approximately 500,000 cells were plated and used for immunolabeling. Three antibody panels were used per sample (**Supplemental Table 1**). Cells were blocked with 5 uL of normal dog serum (panel 1 & 2) or 5 uL of normal donkey serum (panel 3) for 15 minutes on ice (Jackson Laboratory; Bar Harbor, ME). Primary antibodies diluted in FACS buffer (5% FBS plus 0.1% sodium azide in PBS) were added for 30 minutes (only mouse anti-CADO48a for panel 3), then for panel 3 a donkey anti-mouse secondary antibody (30 minutes) followed by strepdavidin-Qdot800 (15 minutes) and directly conjugated antibodies were added (30 minutes) on ice. Cells were washed twice with FACS between each labeling step. After completion of immunolabeling, 5 uL of 7-aad were added to each sample then run on a Beckman Coulter Gallios 3-laser flow cytometer. A total of 150,000 events were targeted during data acquisition.

## Results

### Establishment of a healthy canine leukocyte reference database

The first objective of this study was to establish a comprehensive canine reference leukocyte database that can be used to further define immune cell transcriptomes and be available for use by other research groups. To establish the reference dataset, we obtained a total of 32,028 cells from 3 male and 4 female middle-aged, clinically healthy dogs (**Table 1**). The average number of cells collected per dog was 4,575 and on average each cell was sequenced to a depth of 60,686 reads per cell.

**Table 1:** Dog demographics.

Group	ID	Sex	Age	Breed
Healthy	H_1	FS	8.3	Mixed
	H_2	MC	8.4	Australian Shepherd
	H_3	FS	6.9	Standard Poodle
	H_4	MC	7.4	Beagle
	H_5	FS	8.8	Mixed
	H_6	FS	7.7	Labrador Retriever
	H_7	MC	7.7	Newfoundland
Osteosarcoma	OS_1	MC	10	Labrador Retriever
	OS_2	FS	9	Saint Bernard
	OS_3	FS	6.8	Great Dane
	OS_4	MC	11.5	Catahoula
	OS_5	MC	12.1	Rottweiler
	OS_6	MC	6.8	German Shepherd
	OS_7	FS	7.3	Great Dane
	OS_8	FS	5.7	Staffordshire Terrier mix
	OS_9	MI	7.8	Bernese Mountain Dog/Great Pyrenees
	OS_10	MI	10.4	Saint Bernard

**Figure 1:**
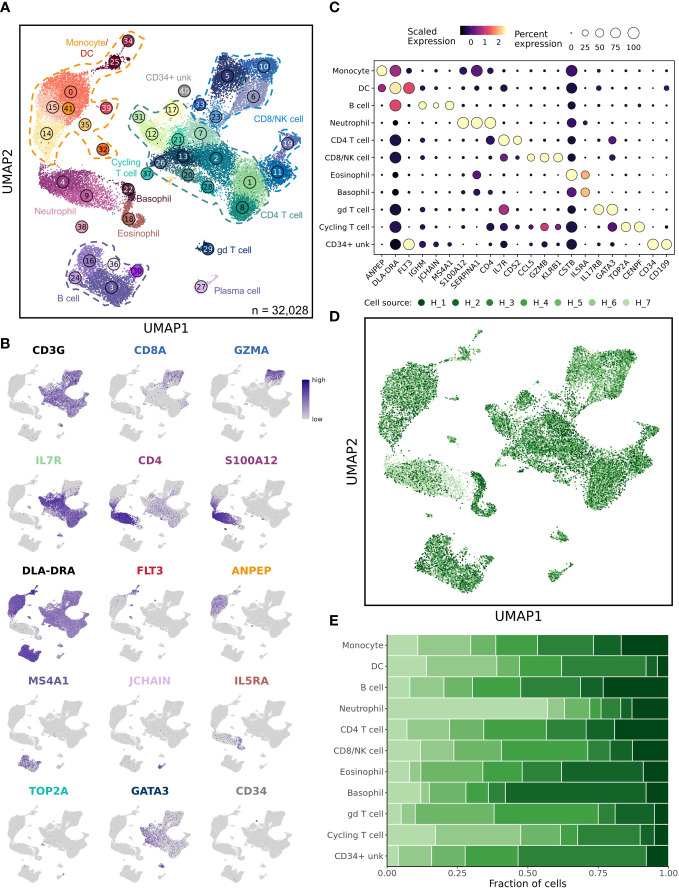
Unsupervised clustering reveals 42 unique cell populations in healthy dog leukocytes. **(A)** UMAP representation depicting the unsupervised clustering results of 32,028 leukocytes from 7 healthy dogs. Major immune cell populations are labeled on the plot (CD34+ unk = CD34+ unclassified; gd = γδ) and each number corresponds to a unique cluster, with numbers increasing as cluster size becomes smaller. **(B)** Feature plots depicting expression of stereotypic genes used to define cell populations. **(C)** Dot plots illustrating scaled gene expression grouped by major immune cell populations. **(D)** UMAP representation of healthy canine leukocytes down sampled to obtain equal sampling of each dog and colorized by biological replicate (n = 7 dogs). **(E)** Stacked bar graph depicting contribution of each sample to every cell type.

In total, 42 unique cell clusters were identified with major immune cell populations clustering in distinct regions of the uniform manifold approximation and projection (UMAP) plot (**Figure 1A**). Cell identities were assigned based on feature plots using stereotypic markers reported in the literature (**Figures 1B, C**) (16). To further support our classifications, we used reference mapping to human databases (**Supplemental Figures 1A–E**). Unexpectedly, cell classifications based on human references were highly variable and largely ineffective at assigning cell identities, especially to CD8 T cell, NK cell, and myeloid cell populations. This variability is likely the result of incomplete canine genome annotation and distinct cellular transcriptomes between species. Following cell classification, we evaluated the relative contribution of each sample to every cluster (**Figures 1D, E**; **Supplemental Figure 2A**). This analysis revealed that the dataset was well integrated without overt batch effects, and most dogs contributed equally to each cluster. The exceptions were within two neutrophil clusters, Clusters 4 and 9, which were largely composed of cells from healthy dog 7. In mice and humans, neutrophils are reported to have a cellular density that is too dense to be isolated in the polymorphonuclear cell (PBMC) layer when completing density centrifugation (24). However, consistent with previous reports, we present evidence that a sizeable population of canine neutrophils have a density that allows them to be collected with PBMC isolation (25). Additionally, there is evidence of marked inter-sample variability in the number of neutrophils isolated through density centrifugation, which can alter overall cellular proportions in a given sample.

Although UMAP plots do not perfectly reconstruct the relative spatial relationship between cells, we noted that myeloid cells largely clustered together, while T cells and B cells clustered in different regions of the plot, indicating the data clustered in a biologically relevant manner. Interestingly, there was one rare myeloid cell cluster (Cluster 38) which clustered in a distant location from the other neutrophils and lacked CD4 expression. Additionally, one T cell population (Cluster 29) was plotted in a distant location relative to other T cells; this population was identified as a gd (γδ) T cell population based on GATA3 and TRDC expression (**Figure 1B** and **Supplemental Figure 1F**). Another interesting observation was that the CD8 T cell populations clustered in two separate regions of the UMAP, with naïve CD8 T cells appearing to be more similar to naïve CD4 T cells than effector CD8 T cells. This clustering of T cells is likely a result of naïve CD8 T cells lacking cytotoxic properties that arise following interaction with their cognate antigen.

Upon classification of all cell populations identified in the 7 healthy dogs, we generated gene lists that define each cell population and provide these lists in **Supplemental Data 1**. In summary, we present a road map of healthy canine leukocytes and provide transcriptomic signatures for each distinct immune cell type that can be applied to study canine immunology as well used as a reference for deconvolution of bulk RNA sequencing data. Next, we applied the reference to investigate how the presence of osteosarcoma alters leukocyte transcriptional programs.

### Comparison of healthy and osteosarcoma affected canine leukocytes

**Figure 2:**
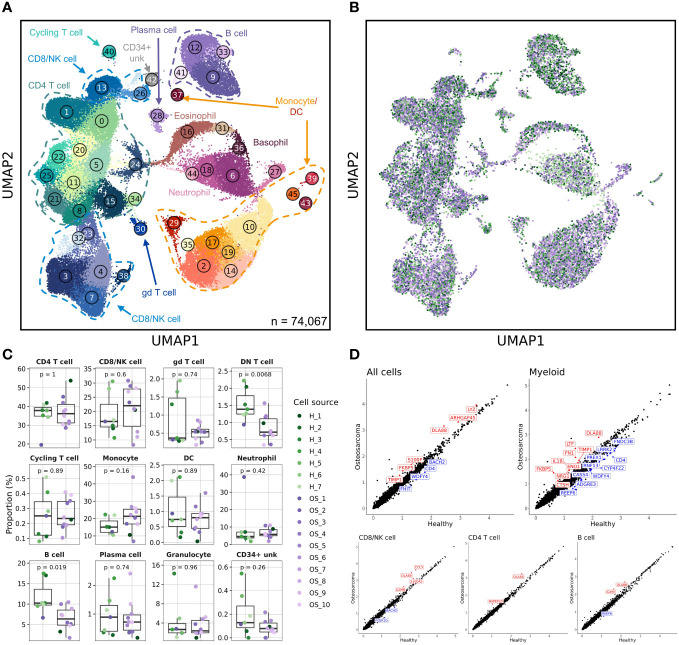
Canine osteosarcoma contributes to reductions in the relative abundances of double negative T cells and B cells, while myeloid cells contribute to most transcriptomic aberrations. **(A)** UMAP representation depicting the unsupervised clustering results of 74,067 leukocytes from 7 healthy and 10 osteosarcoma dogs (CD34+ unk = CD34+ unclassified). **(B)** UMAP representation of healthy (n = 7) and osteosarcoma (n = 10) canine leukocytes down sampled to obtain equal sampling of each dog and colorized by biological replicate. Legend is shared with **(C)**. **(C)** Box plots quantifying changes in cell abundances between healthy and cancer dogs (Granulocyte = Eosinophil and Basophil clusters; DN T cell = Cluster 26 of CD8/NK T cell group). P values were obtained using a two-sided Wilcoxon Rank Sum test. **(D)** Scatter plots comparing average feature expression in osteosarcoma (y-axis) verses healthy cells (x-axis) with all (74,067) cells and 4 of the major immune cell populations, with labeled features significantly altered, as determined using a Wilcoxon Rank Sum test.

We isolated circulating leukocytes from 6 male and 4 female middle-aged tumor bearing dogs diagnosed with osteosarcoma (OS) (**Table 1**; **Supplemental Table 2**). The cells obtained from the cancer burdened dogs were integrated with the 7 healthy samples to obtain a complete dataset of 74,067 cells. Similar to the unsupervised clustering of the healthy samples, we identified 46 unique clusters with the major immune cell types apparent (**Figure 2A**). Evaluation of data integration revealed uniform distribution of cells across all samples, except for clusters 9, 12, and 26 (**Figures 2B, C** and **Supplemental Figure 2B**).

Clusters 9 and 12 (B cell populations) and Cluster 26 (a double negative [CD3^+^/CD4^-^/CD8^-^] T cell population) were found to be underrepresented in the OS dogs. Interestingly, there has been one report of B cell and double negative (DN) T cell reductions in the peripheral blood of non-small cell lung cancer patients (26). While these findings agree with our data, other reports have documented reductions in B cell and DN T cell abundances with age (27, 28). Therefore, to investigate further, we compared the relative contribution of cell types in the middle-aged OS (aged 5-8 years old) and old OS dogs (aged 9-12 years old). We found no difference in B cell or DN T cell abundances between middle-aged and old OS dogs, while comparisons between healthy and middle-aged OS dogs also suggested no differences in abundance (**Supplemental Figure 3**). Therefore, due to the limited sample size and previous reports of age-dependent B cell and DN T cell reductions, we believe the observed reductions in our dataset are likely in part due to age and not a direct effect of OS.

Aside from changes in cell abundances, we also observed that the CD4^-^ neutrophil population identified in the healthy dataset (Cluster 38 in **Figure 1A**) shifted its position on the UMAP to be closer to other neutrophil populations (Cluster 27 in **Figure 2A**). This shift is attributed to the addition of cell numbers increasing the sample size and enabling the UMAP dimension reduction to better reconstruct the relative position on this cell population. While this population visually appeared to be expanded, statistically, there was no difference in abundance when evaluating the percentage out of total leukocytes using the clustering results from all immune cells.

Finally, we evaluated differentially expressed genes in all cells and each of the major immune cell populations (**Figure 2D**). This analysis indicated that several features were overrepresented in dogs with OS, with the most prominent differences arising from changes to myeloid cell gene expression. In particular, we observed IL1B and LTF upregulation in dogs with OS and a slight reduction of CD4 expression in dogs with OS. To further evaluate transcriptional differences in health and diseased states, we subset the database on each major cell population and completed independent unsupervised clustering to obtain greater resolution.

### CD8 T cells, double negative T cells, and NK cells

**Figure 3:**
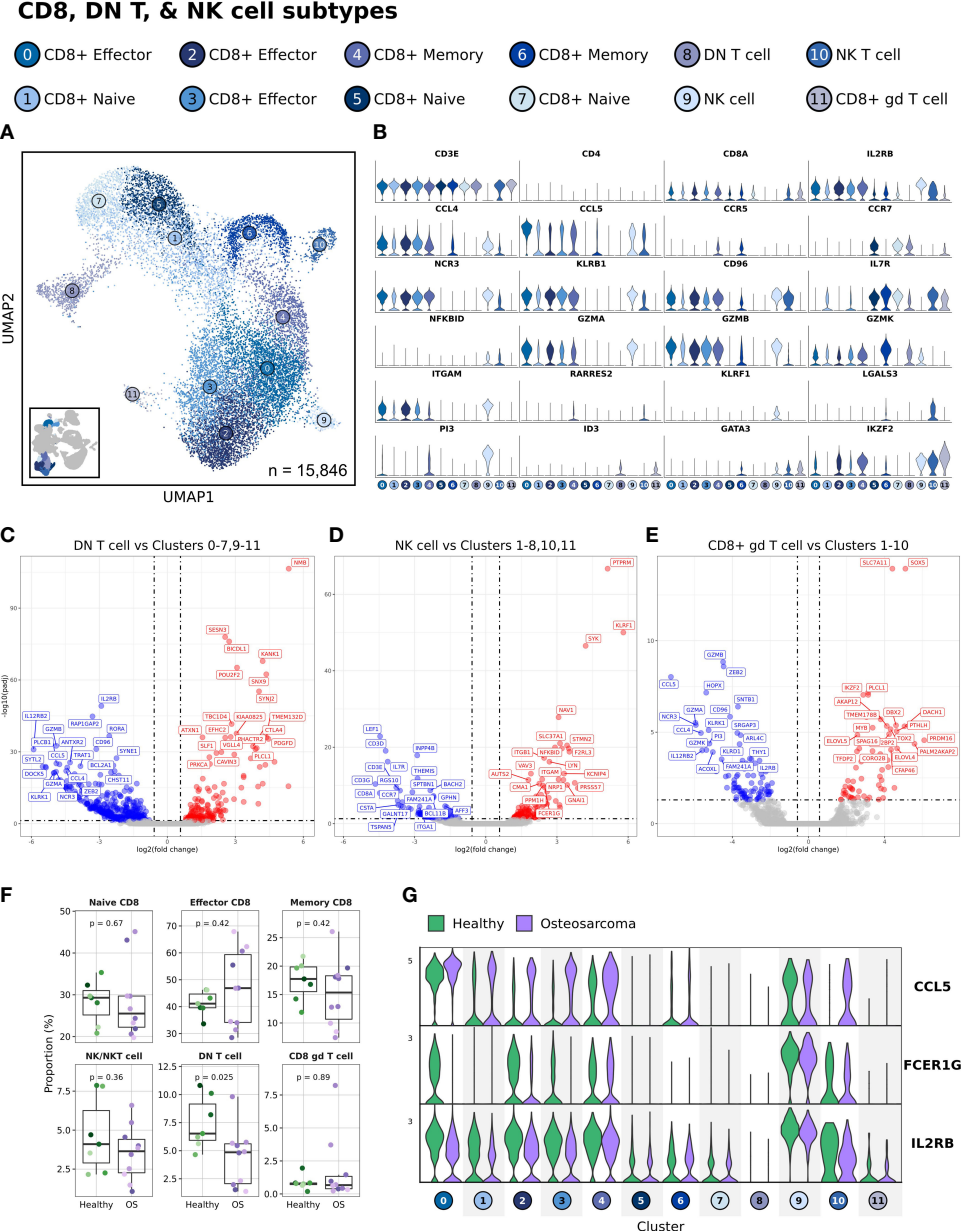
Double negative T cell abundances are reduced in dogs with cancer, while CCL5 expression is increased in effector CD8 T cell clusters. **(A)** UMAP representation depicting the unsupervised clustering results of 15,846 leukocytes (n = 7 healthy and 10 osteosarcoma dogs). **(B)** Violin plots depicting key feature expression for each cytotoxic cell cluster. Axis scales are fixed across all features. **(C-E)** Volcano plots depicting differential gene expression when comparing DN T cell, NK cell, and CD8^+^ gd T cell populations to all reaming cell clusters. **(F)** Box plots quantifying changes in cell cluster abundances (percentage of total cells, n = 15,846) between healthy and cancer dogs. P values were obtained using a two-sided Wilcoxon Rank Sum test. **(G)** Violin plots of three features (CCL5, FCER1G aka Fc Epsilon Receptor Ig, and IL2RB aka CD122) identified as increased (CCL5, FCER1G) or decreased (IL2RB) in at least one cell cluster.

In depth analysis of CD8 T cell, double negative T cell, and NK cell populations consisted of 15,846 cells with unsupervised clustering revealing 12 distinct clusters. Within this major immune cell population, we identified DN T cells, naïve CD8 T cells, effector CD8 T cells, memory CD8 T cells, NK cells, and NK T cells (**Figures 3A, B** and **Supplemental Figures 4A–C**). Unexpectedly, we also identified a population of CD8+ gd T cells (Cluster 11) based on TRDC expression (**Supplemental Figure 4D**). Of note, we found TRDC expression to extend outside of the CD8+ gd T cell cluster, with intermittent gene expression in effector and naïve CD8 T cells as well as uniform expression in NK cells. In addition to manual and algorithmic classification methods, we completed differential gene expression analysis to determine which features define key populations.

The results of differential gene expression using pseudobulk analysis from comparing one cluster to all other cells allowed for a complete description of the features that define each cell type. We found that the DN T cell cluster had reduced expression of cytotoxic genes (GZMB, NCR3, and KLRK1) and increased CTLA4 expression relative to all other cytotoxic cells (**Figure 3C** and **Supplemental Data 3**). These gene patterns suggest the population may have reduced cytotoxic potential and may exhibit immune suppressive properties. When completing the same analysis on the NK cell population we found a reduction in T cell markers (CD3E, CD3G, CD8A) with an increase in certain cytotoxic features (GZMA, CD96 and KLRF1) relative to other cells (**Figure 3D** and **Supplemental Data 3**). Finally, we applied this approach to further investigate Cluster 11, a CD8^+^ gd T cell population (**Figure 3E** and **Supplemental Data 3**). This analysis revealed that several cytotoxic features were downregulated (GZMA, GZMB, GZMK, KLRK1, and NCR3) while also indicating IKZF2, SOX5, and ELOVL5 were upregulated relative to other cytotoxic cells.

With cell classifications established, we next investigated the earlier observation that DN T cells were reduced in dogs with OS. To complete this analysis, we evaluated the cellular abundances as a percentage of the total cells (15,846 cells) in this immune cell subset and the results were consistent with the earlier approach (**Figure 3F**). Despite the marked reduction in DN T cells, we believe the decrease is in part due to age and should be interpreted cautiously. Unexpectedly, there were minimal transcriptomic differences when comparing healthy and OS CD8 T cell populations, but we consistently observed an increase in CCL5 expression on effector CD8 T cells in OS dogs (**Figure 3G**). Although less pronounced, we also identified decreases in FCER1G and IL2RB on effector CD8 T cell populations in dogs with OS. Together, these transcriptomic changes suggest there may be altered T cell recruitment signals in effector CD8 T cells of dogs with OS.

### CD4 T cells

**Figure 4:**
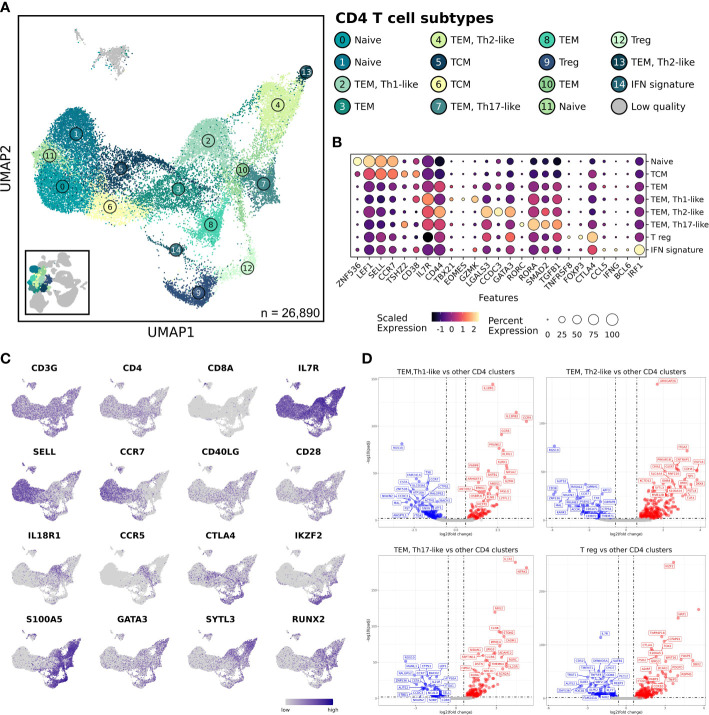
Analysis reveals 4 major CD4 T cell subtypes that were unaltered when comparing between healthy and osteosarcoma leukocytes. **(A)** UMAP representation depicting the unsupervised clustering results of 26,890 leukocytes (n = 7 healthy and 10 osteosarcoma dogs). **(B)** Dot plots illustrating gene expression by major CD4 T cell classification. Cell sub-classifications were collapsed to obtained 1 cell type group. **(C)** Feature plots depicting expression of stereotypic features used to define CD4 T cell populations. **(D)** Volcano plots depicting differentially expressed gene that were identified when comparing TEM Th1-like, Th2-like, Th17-like, and T regulatory cell clusters to all other CD4 T cell clusters. TCM, T central memory; TEM, T effector memory; Treg, regulatory T cell; IFN, interferon.

To continue a deeper investigation of major immune cell populations, we next focused our analysis on the most heterogenous group of cells: the CD4 T cells. Following independent re-clustering, an additional 3 clusters were revealed which together indicated the presence of 15 transcriptionally unique CD4 T cell populations (**Figure 4A** and **Supplemental Figure 5A**). One additional cluster was determined to be of poor quality and is depicted (grey) but was excluded from downstream analysis. Comparisons between healthy and OS CD4 T cells revealed no apparent aberrations in cell abundances or transcriptomes (**Supplementary Figure 5B**). Therefore, our analysis of CD4 T cells focused on describing the transcriptomic signature of each unique CD4 T cell population.

We used stereotypic markers and reference mapping to assign cell identities (29, 30). This enabled the identification of naïve, central memory (TCM), effector memory (TEM), T regulatory (Tregs), and interferon (IFN) signature CD4 T cell populations (**Figures 4B, C**). The IFN signature cell type (Cluster 14) was defined by high expression of features associated with IFN response pathways when completing gene set enrichment analysis (**Supplemental Figure 5C**) (31). The heterogeneity within the TEM clusters was further investigated and revealed the presence of Th1-like, Th2-like, and Th17-like clusters (**Supplemental Figure 5D**) (17). Next, we used pseudotime trajectory analysis to investigate how the CD4 T cell populations were related. To complete this analysis, we established the naïve CD4 T cell Cluster 0 as the root node, then used Slingshot to infer how cell transcriptomes progressed (19). The analysis indicated several branchpoints, with 4 major lineages identified (**Supplemental Figures 5E, F**). All lineages began at the assigned naïve CD4 T cell cluster (Cluster 0), progressed through a TCM cluster (Cluster 5), then through a TEM, and finally diverged toward an endpoint. Lineage 1 was determined to represent the progression of Th1-like cells, lineage 2 tracked the Th2-like progression, lineage 3 illustrated the Th17-like progression, and lineage 4 represented the progression of Tregs. Finally, for each of the four-lineage endpoints we completed differential gene expression analysis to further define the cell populations (**Figure 4D** and **Supplemental Data 3**). Each population had upregulation of canonical features (Th1 = IL18R1/TBX21; Th2 = GATA3; Th17 = RORA/RORC, Treg = CTLA4/FOXP3) which acted to further validate cell classifications and provide novel markers to distinguish each cell type (29, 32). In summary, we observed minimal aberrations in cell abundance or transcriptomes of CD4 T cell population in OS dogs but were able to identify 4 major lineages which provides insight into CD4 T cell biology in dogs.

### Myeloid cells

**Figure 5:**
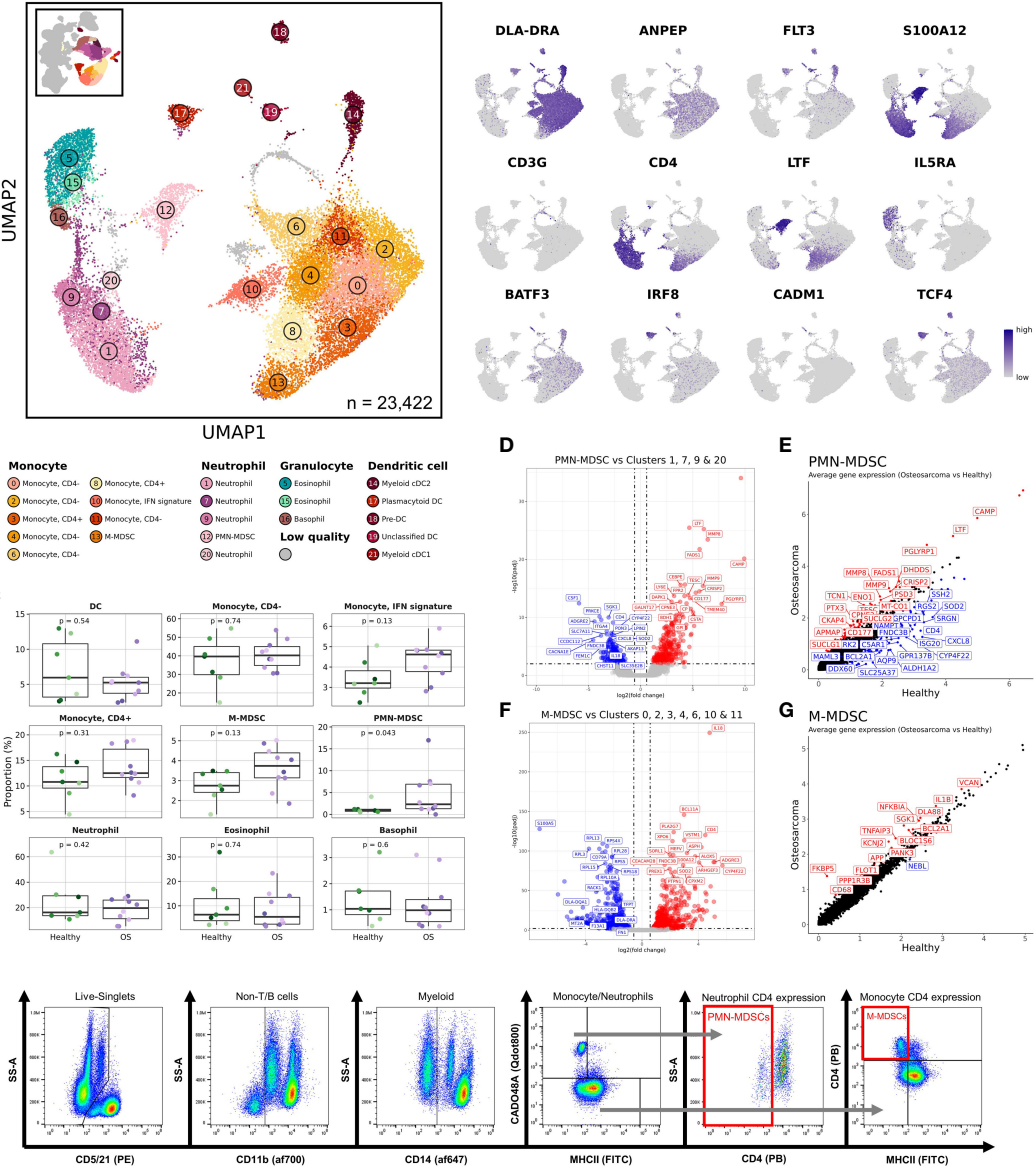
CD4 negative neutrophils are expanded in dogs with osteosarcoma and transcriptomically resemble polymorphonuclear myeloid-derived suppressor cells. **(A)** UMAP representation depicting the unsupervised clustering results of 23,422 myeloid cells (n = 7 healthy and 10 osteosarcoma dogs). **(B)** Feature plots depicting expression of key features used to define myeloid cell populations. **(C)** Box plots for each major myeloid cell population quantifying changes in cell abundances between healthy and cancer dogs. P values were obtained using a two-sided Wilcoxon Rank Sum test. **(D)** Volcano plots depicting differentially expressed genes identified when comparing the PMN-MDSC cluster (Cluster 12) to all other neutrophil clusters (Clusters 1,7,9,20). **(E)** Scatter plot comparing average feature expression in osteosarcoma (y-axis) verses healthy (x-axis) cells within the PMN-MDSC cluster. **(F)** Volcano plots highlighting differentially expressed genes identified when comparing the M-MDSC cluster (Cluster 13) to all other monocyte clusters (Clusters 0,2,3,4,6,8,10,11). **(G)** Scatter plot comparing average feature expression in osteosarcoma (y-axis) verses healthy (x-axis) cells within the M-MDSC cluster. **(H)** Representative flow cytometry gating strategy used to identify PMN- and M-MDSC populations.

The next major immune cell population we analyzed consisted of monocytes, dendritic cells, neutrophils, basophils, and eosinophils. Through completion of independent re-clustering, we identified 22 high quality myeloid cell populations and 2 low quality clusters that were excluded from downstream analysis (**Figure 5A** and **Supplemental Figure 6A**). Monocytes were the most heterogenous cell type with 9 unique clusters, followed by 5 dendritic cell clusters, 5 neutrophil clusters, 2 eosinophil clusters, and 1 basophil cluster. Stereotypic features were then used to confirm cell identities (**Figure 5B**) (4, 16, 33). Although CD4 is reported to be expressed on canine neutrophils by flow cytometry, we identified a distinct neutrophil population that lacks CD4 expression (Cluster 12) (34). This unique neutrophil population appeared to be immature with immune suppressive gene expression patterns and was assigned an identity of polymorphonuclear myeloid-derived suppressor cells (PMN-MDSCs). When annotating the monocyte populations, we intended to define subpopulations by CD14/CD16 expression, as used for humans and mice. However, both CD14 and CD16 lack annotation in the primary canine reference genome (CanFam3.1) used in this study. As an alternative approach, we used CD64 (FCGR1A), MHCII (DLA-DRA), and CD86 to characterize to evaluate these cell populations in the context of human nomenclature (**Supplemental Figure 6B**) (35). We determined that CD4^+^ monocytes and M-MDSCs resembled classical monocytes (CD14^+^/CD16^-^), while CD4^-^ monocytes resembled non-classical monocytes (CD14^-^/CD16^+^). Despite some overlap in nomenclature, the human classifications largely did not translate to our dataset, so we used CD4 expression to define canine monocyte populations.

To investigate how cell abundances were altered by OS, we compared the cellular frequencies of major myeloid cell sub-populations (**Figure 5C**). Consistent with the human literature, this analysis revealed PMN-MDSCs were expanded in dogs with OS (36, 37). While not statistically significant as a percentage of myeloid cells, we observed a statistically significant increase in monocytic (M-) MDSCs (Cluster 13) when evaluating the cells as percentage of total leukocytes (**Table 2**). Differential gene expression analysis between OS and healthy dogs within monocytes exhibiting an IFN signature (Cluster 10) revealed a reduction in IFN-related gene expression in dogs with OS (**Supplemental Figure 6C/D**). This interesting change in gene expression indicates that dogs with OS may have a reduced IFN response potential in this subset of monocytes.

**Table 2:** Canine leukocyte high-resolution cellular composition.

		All	Healthy	Osteosarcoma	P value*
CD8 T cell		19.9 ± 7.6	18.4 ± 7.2	21 ± 8	0.601
CD8+ Naive		6.1 ± 2.5	5.9 ± 2.8	6.2 ± 2.4	0.536
CD8+ Effector		10.2 ± 5.6	8.7 ± 3.9	11.3 ± 6.5	0.669
CD8+ Memory		3.4 ± 1.3	3.6 ± 1.3	3.2 ± 1.4	0.813
CD8+ gd T cell		0.2 ± 0.2	0.2 ± 0.1	0.3 ± 0.3	0.962
CD4 T cell		36.2 ± 7.6	35 ± 7.5	37 ± 7.9	1
CD4+ Naive		10.3 ± 3.4	10.4 ± 3.1	10.2 ± 3.8	0.962
CD4+ TCM		5.3 ± 1.6	5 ± 1.3	5.4 ± 1.9	1.000
CD4+ TEM		7.4 ± 2.1	7.5 ± 2.2	7.3 ± 2	0.813
CD4+ TEM, Th1-like		3.8 ± 1.8	3.4 ± 1.9	4 ± 1.7	0.475
CD4+ TEM, Th2-like		4 ± 1.6	3.7 ± 1.4	4.3 ± 1.7	0.740
CD4+ TEM, Th17-like		2.1 ± 0.7	1.8 ± 0.4	2.3 ± 0.8	0.161
CD4+ T reg		3 ± 0.7	2.9 ± 0.5	3 ± 0.9	1.000
CD4+, IFN signature		0.3 ± 0.1	0.3 ± 0.1	0.4 ± 0.1	0.813
Monocyte		18.5 ± 8.5	15.1 ± 4.1	20.8 ± 10.1	0.088
Monocyte, CD4-		12.2 ± 5.8	10.2 ± 3.2	13.6 ± 7	0.230
Monocyte, CD4+		4 ± 2.2	3.1 ± 1.1	4.7 ± 2.6	0.230
M-MDSC		1 ± 0.4	0.8 ± 0.2	1.2 ± 0.4	0.019
Monocyte, IFN signature		1.3 ± 0.6	1.1 ± 0.8	1.4 ± 0.5	0.193
Dendritic cell		1.8 ± 1	2 ± 1.3	1.6 ± 0.8	0.813
Pre-DC		0.3 ± 0.2	0.3 ± 0.3	0.2 ± 0.2	0.536
Myeloid cDC1		0.1 ± 0.1	0.1 ± 0.1	0.1 ± 0.1	0.669
Myeloid cDC2		0.9 ± 0.5	0.9 ± 0.5	0.9 ± 0.5	1.000
Plasmacytoid DC		0.3 ± 0.3	0.4 ± 0.4	0.3 ± 0.2	0.601
Unclassified DC		0.1 ± 0.1	0.2 ± 0.2	0.1 ± 0.1	0.962
Granulocyte		11.1 ± 8.1	13.1 ± 12.2	9.8 ± 3.4	1
Neutrophil		6.9 ± 7.6	9.2 ± 11.5	5.4 ± 2.9	0.6691
PMN-MDSC		1 ± 1.2	0.5 ± 0.8	1.4 ± 1.4	0.0431
Eosinophil		2.8 ± 3	3 ± 3.5	2.6 ± 2.7	0.8125
Basophil		0.4 ± 0.3	0.4 ± 0.2	0.4 ± 0.3	0.7396
B cell		9.5 ± 4.5	12.6 ± 4.4	7.4 ± 3.3	0.07
Immature B cell		0.8 ± 0.5	1.1 ± 0.5	0.6 ± 0.4	0.088
Naive B cell		5.4 ± 2.6	7 ± 2.5	4.3 ± 2.1	0.043
Class switched B cell		1.7 ± 0.8	2.4 ± 0.7	1.2 ± 0.5	0.005
Activated B cell		0.7 ± 0.9	1.2 ± 1.2	0.4 ± 0.3	0.014
Plasma cell		0.9 ± 0.6	1 ± 0.7	0.8 ± 0.6	0.887
Miscellaneous		3 ± 1.2	3.7 ± 1.4	2.4 ± 0.7	0.025
DN T cell		1.1 ± 0.5	1.4 ± 0.4	0.8 ± 0.3	0.005
gd T cell		0.7 ± 0.5	0.9 ± 0.7	0.5 ± 0.2	0.740
NK cell		0.5 ± 0.4	0.6 ± 0.6	0.4 ± 0.3	0.364
Cycling T cell		0.3 ± 0.1	0.3 ± 0.2	0.3 ± 0.1	0.887
NK T cell		0.3 ± 0.2	0.3 ± 0.1	0.4 ± 0.2	1.000
CD34+ unclassified		0.1 ± 0.1	0.2 ± 0.2	0.1 ± 0.1	0.261

*P value is the exact two-sided P value obtained by a Wilcoxon Rank Sum test comparing Osteosarcoma column to Healthy.

We next evaluated differentially expressed genes to determine the transcriptomic signatures for the suspected PMN-MDSC cluster (**Figure 5D** and **Supplemental Data 3**). We found stereotypic PMN-MDSC features (CD177, LTF, CAMP, MMP9, and MMP8) to be upregulated in the PMN-MDSC cluster relative to the other neutrophil clusters (Clusters 1, 7, 9, & 22) (38). This analysis supported the classification of Cluster 12 as PMN-MDSCs. To determine if there were transcriptomic differences between healthy and OS PMN-MDSCs, we used a Wilcoxon Rank Sum test to identify differentially expressed genes and found the expression of CD4, ISG20, and CXCL8 to be higher in cells from healthy dogs (**Figure 5E**). These findings suggest that either the healthy dog PMN-MDSCs are distinct from the OS derived PMN-MDSCs, or more likely, that normal neutrophils were misclassified during unsupervised clustering. Another immune suppressive cell population previously identified to be expanded in cancer patients are M-MDSCs (39). We suspected that the CD4^+^ monocyte population, Cluster 13, represented M-MDSCs. When evaluating differentially expressed genes between Cluster 13 and the remaining monocytes (Clusters 0, 2, 3, 4, 6, 10, and 11), we found down regulation of ribosomal transcripts (RPS/RPL) and MHCII (DLA-DRA) features and upregulation of CD4, S100A12, and IL18 (**Figure 5F** and **Supplemental Data 3**). While not definitive, this expression profile supports the classification of Cluster 13 as M-MDSCs, while also providing the gene signature of Cluster 13 (40, 41). Investigation of transcriptomic differences in disease revealed subtle changes in gene expression, with higher IL1B and lower DLA-DRA in the dogs with OS within the M-MDSC cluster (**Figure 5G**).

Following the identification of M-MDSC and PMN-MDSC populations, we developed a clinically accessible flow cytometry-based assay to monitor MDSC populations in dogs with cancer (**Figure 5H**, **Supplemental Figure 6E** and **Supplemental Table 1**). The assay builds upon previously proposed canine MDSC immunolabeling protocols, but includes an anti-CD4 antibody to further distinguish MDSCs from their normal counterparts (9, 42, 43). This assay is intended to be employed in the investigation of prognostic correlates and further study of canine MDSC biology.

### B cells and miscellaneous cell types

The final immune cell population analyzed was B cells. We were able to identify immature, naïve, activated and class-switched B cells as well as a cluster of plasma cells (**Supplemental Figures 7A–C**). We found minimal differences within B cells when comparing between the cell abundances and transcriptomes of OS verses healthy dogs (**Supplemental Figure 7D**). Despite the lack of differences between healthy and diseased states, the data presented here provide transcriptomic signatures for B cell subtypes not previously resolved using traditional characterization methods in dogs.

In addition to the immune cell populations discussed above, we reported the presence of gd T cells, cycling T cells and a CD34+ unclassified population. Initially, we believed the CD34+ unclassified cluster to be common myeloid progenitors. Upon closer examination, we noted the population expressed endothelial cell markers (CD109) (44). Therefore, we state their CD34 positivity, but refrain from assigning a further identity due to the small representation of the cluster and conflicting gene signatures. Ultimately, investigation into each miscellaneous population did not reveal the presence of OS mediated changes, so we solely present their transcriptomic signatures as a resource for future researchers.

### Summary of the high-resolution canine leukocyte landscape

Lastly, the cell classifications determined through analysis of individual immune cell populations were compiled to present relative abundances of total circulating leukocytes. We identified 36 unique cell populations believed to each represent a biologically relevant subset of canine immune cells (**Table 2** and **Supplemental Figure 8A/B**). Furthermore, we provide the complete transcriptomic signatures for each cell type and present a summary table with short gene lists for each population (**Supplemental Data 1**, **2**, **4**, **Table 3**). Comparisons between healthy and OS dogs revealed both PMN-MDSCs and M-MDSCs were expanded in dogs with OS when evaluated as a percentage out of all leukocytes. Additionally, DN T cells, naïve B cells, class-switched B cells, and activated B cells were found to be reduced in dogs with OS, but we were unable to determine if this is a result of age or cancer.

**Table 3:** Transcriptional signatures of canine leukocytes.

Cell Type		Marker
CD8 T cell		
CD8+ Naive		ITGA1, LEF1, PTGDR, IL2RB, ADGRG1, NBEA
CD8+ Effector		CCL5, TRPM3, IL12RB2, GZMB, KLRB1, GZMA, NCR3, IL2RB, KLRD1, CD96
CD8+ Memory		GZMK, GZMB, PI3, BTBD11, CTSW, CCR5, CCL4, KLRG1, FASLG
CD8+ gd T cell		PTHLH, IGF2BP2, ABTB2, AKAP12, SOX4, CTSW, SLC16A10, PXT1, ZNRF3, SULT2B1
CD4 T cell		
CD4+ Naive		LEF1, CSTA, RGS10, ZNF536, CCR7, COL6A5, LTB, TNFSF8
CD4+ TCM		LEF1, TSHZ2, CD52, CCR7, IL7R, CTPS1, EFHC2, CARMIL1
CD4+ TEM		IL7R, SLC9A9, ICOS, MAF, CD28, SKAP1, CD40LG
CD4+ TEM, Th1-like		IL7R, PTPN13, IL18R1, CD28, RCAN2, CCR9, CCR5, IL12RB2, CD52, PRUNE2
CD4+ TEM, Th2-like		RNF220, ITGA2, GATA3, CCDC3, LGALS3, PTPN13, S100A2, PPEF1, CMA1
CD4+ TEM, Th17-like		NTRK2, PTPN13, ADAM12, NRG2, RGS17, DNAH8, CCR6, NPAS2, RORA, LTBP1
CD4+ T reg		IKZF2, CTLA4, RGS1, ICOS, IL2RA, CD28, ZNF831
CD4+, IFN signature		CXCL10, IFI44, OAS1, ISG15, IFI44L, IFGGB2, CTLA4, STAT1, DDX58, XAF1
Monocyte		
Monocyte, CD4-		LYZ, BPI, LRMDA, MT2A, F13A1, FN1, NRG1, CCDC88A, CD83, RETN
Monocyte, CD4+		IL1B, MAFB, NFKBIA, CXCL8, FN1, BLOC1S6, CD83, S100P, BPI, NRG1
M-MDSC		IL18, IL1B, LTF, MEFV, KCNJ2, CPXM2, S100A12, STEAP4, CSF3R, IL31RA
Monocyte, IFN signature		RSAD2, OAS1, OAS2, DDX58, HERC6, OAS3, RTP4, EIF2AK2, IFIT2
Dendritic cell		
Pre-DC		FGF12, GPHA2, MTUS2, FCER1A, PLCE1, PTPRS, IGF1, NECTIN1, IL3RA, AK8
Myeloid cDC1		ZNF366, SDC2, DISC1, ECRG4, TMEM163, RIMS2, KIT, OTOF, RTKN2, RAB7B
Myeloid cDC2		PKIB, CD300H, SDC2, CD1C, NCAM2, CD86, BATF3, ZNF366, PID1, ECM1
Plasmacytoid DC		COBLL1, RAB3C, IGF1, FCER1A, RYR1, PRKG1, CCND1, STYXL2, ANK1, OCIAD2
Unclassified DC		PLCB4, ZNF366, KCNK13, STRIP2, SDC2, OTOF, HACD1, C5, SLC8A1, CNTLN
Granulocyte		
Neutrophil		S100A12, CD4, SERPINA1, SGK1, S100A8, ALDH1A2, FNDC3B, GGH, SRGN, IL1R2
PMN-MDSC		CAMP, PGLYRP1, CRISP2, MMP9, MMP8, TCN1, CD177, LTF, FADS1, S100A12
Eosinophil		C30H15orf48, TGM2, DACH1, PADI3, SMPD1, CA8, IL5RA
Basophil		DACH1, CA8, IL5RA, DAPK2, TGFA, ANKRD33B, HK2, PRR5L
B cell		
Immature B cell		SYT1, PAX5, VPREB3, ERC2, TMTC2, KLHL14, F8, TEX9, TDRP, ADGRF1
Naive B cell		TNFRSF13C, BANK1, HTR1F, PAX5, EBF1, BTLA, NRIP1, ADAM9
Class switched B cell		TNFRSF13C, GOLM1, BANK1, BTLA, EBF1, DYNC1I1, MTMR2, PAX5
Activated B cell		IGKC, CACNB2, PAX5, TNFRSF13C, IGHM, RASGRF2, AOX2, BCAR3, ADAM32
Plasma cell		JCHAIN, MZB1, TXNDC5, LMAN1, FKBP11, LAP3, DERL3, CCR10, MKI67, TNFRSF13B
Miscellaneous		
DN T cell		KIAA0825, TMEM132D, KANK1, NMB, CTLA4, SYNJ2, BICDL1, SLF1, ID3, KIAA1549
gd T cell		PARD3B, RHEX, IL17RB, CDH4, GATA3, FAT1, TOX2, ADARB1, ZNF683, TGFBR3
NK cell		KLRF1, STMN2, PAX4, NCR3, F2RL3, CD96, IL2RB, IGSF3, FREM1, FASLG
Cycling T cell		TOP2A, MKI67, RRM2, H1-5, DIAPH3, TK1, KIF11, TPX2, ASPM
NK T cell		GPA33, TGFBR3, KLRK1, CD96, SYTL2, MOV10L1, SLA2, DSTN, RARRES1
CD34+ unclassified		TFPI, ZNF521, CD34, NDST3, GUCY1A1, HPGD, CLEC3B, KIT, CD109, DNTT

Overall, the breakdown of leukocytes was found to be consistent with previously reported values determined using flow cytometry (45). We used paired flow cytometry data to confirm the relative distribution of major immune cell types. The comparison of cellular percentages between single-cell RNA sequencing and flow cytometry revealed a positive correlation indicating consistent identification of immune cells independent of approach (R^2^= 0.635; **Supplemental Figure 8C**). Therefore, the data presented here are largely consistent with traditional classification methods, and act to provide novel insights into the heterogeneity and transcriptomic signatures of canine cell types.

Finally, we integrated our dataset of 7 healthy canine leukocytes with a previously published human reference consisting of 6 healthy adults (18). Cell type homologies between species were then evaluated using hierarchical clustering of SCT normalized data, which evaluated the top 2000 variable features with gene homologies between species. Although the analysis was impacted by differences in the level of cell type annotations between the two studies, the results suggested more similarities than dissimilarities between the species (**Supplemental Figure 9**). For instance, human and canine naïve CD4 T cells, neutrophils, and plasmacytoid DCs paired off 1:1 in terminal clades, suggesting a high degree of similarity. Additionally, human DCs clustered on the same clade as all canine DC subtypes; this was also observed in the case of B cells. Interestingly, we identified subtle differences between species, which included human NK cells clustering with canine CD8 effector T cells and gd T cells from each species clustering into separate clades. Overall, the cross-species analysis emphasizes the similarities in circulating immune cell transcriptional profiles, while also highlighting potential differences between the two species.

## Discussion

In the present study we generated a reference single-cell RNA sequencing dataset using 7 healthy dogs, then applied the database to investigate how osteosarcoma alters immune cell transcriptomes and abundances. Our analysis revealed the heterogenous canine immune landscape and allowed us to define the transcriptomes of distinct immune cell populations. When comparing healthy dogs and dogs with osteosarcoma (OS), we found that a cancer burden contributes to relative increases in the abundances of polymorphonuclear (PMN-) and monocytic (M-) myeloid-derived suppressor cells (MDSCs). Furthermore, we identified that most transcriptomic changes between healthy and OS dogs resulted from changes to myeloid cell populations. In particular, we noted increases in LTF, CAMP, and S100A12 expression which appear to arise from an expansion of MDSCs. Ultimately, the data presented here sheds light on the diversity of canine immune cells and highlights key changes between healthy dogs and dogs with osteosarcoma.

Overall, there were relatively few aberrations in leukocyte populations isolated from dogs with osteosarcoma. Despite this, the observed differences may represent biologically significant changes in dogs with cancer. For instance, MDSCs have been identified as key immune suppressive populations that dampen antitumor immunity and the relative abundances of these cells have been determined to have prognostic correlates (8). Both monocytic and polymorphonuclear MDSCs have been reported in dogs and were identified using flow-cytometric based approaches (9). Our findings support the classifications schemes used, but further suggest that CD4 expression should be considered when evaluating MDSC burden. We report that M-MDSCs exhibit a phenotype of CD5^-^/CD21^-^/CD11b^+^/CD14^+^/CADO48a^-^/MHCII^-^/CD4^+^ and that PMN-MDSCs can be defined as CD5^-^/CD21^-^/CD11b^+^/CD14^int^/CADO48a^+^/MHCII^-^/CD4^-^. Because both MDSCs were determined to express LTF, CAMP, and S100A12 it is possible that the use of bulk RNA sequencing or targeted sequencing approaches may be able to provide indirect assessments of MDSC burdens. Ultimately, further investigation of these cell populations is warranted to determine the clinical relevance of MDSCs in dogs with cancer and other diseases.

Although reductions in B cells and DN T cells were noted, we presented further data to suggest age, in addition to cancer, may be driving this change. Due to this potential confounder and minimal transcriptomic differences between healthy and OS dogs within these cell types, our analysis was limited to characterization of each subtype. Our B cell subtype analysis provides refined B cell classifications in dogs and offers transcriptomic profiles of each population.

Unexpectedly, we did not identify overt OS mediated changes to circulating CD4 T cells. Prior research suggests that regulatory T cells tend to be expanded in tumor bearing humans, mice, and dogs (46, 47). The inability to detect an expansion of Tregs is likely a result of our small sample size and lack of power to detect changes in subtly altered cell abundances. As such, our analysis focused on describing the heterogeneity within CD4 T cell subpopulations to provide a comprehensive description of CD4 T cell subtypes. Key contributions include the identification of four distinct CD4 T cell lineages (Th1-like, Th2-like, Th17-like, and Tregs) and the generation of cell type transcriptomic signatures. Our analysis of CD8 T cells and NK cells revealed that, relative to humans, canine CD8 T cells exhibit gene expression patterns that more closely resemble human NK cells than CD8 T cells. This observation was made early on in analysis when using human classifiers to assign cell identities, and again when we completed a direct comparison to a dataset of healthy adult leukocytes. Thus, our findings suggest canine CD8 T cells exhibit a more innate-like transcriptomic signature relative to their human counterparts. A similar conclusion was recently reported in canine single-cell RNA sequencing of αβ T cells (48), providing further evidence of potential differences between CD8 T cells in dogs and humans.

Outside of the major immune cell populations, we identified a few distinct cell subpopulations, including gd T cells, cycling T cells, and CD34+ unclassified cells. These cell types were considered to be distinct from the major immune cell populations and were left out of independent re-clustering analysis, but their importance should not be discounted given their documented roles in numerous diseases (49). Thus, their characterization may represent a useful reference for future studies.

The cellular annotations proposed in our analysis represent plausible cellular identities that we determined through use of manual and algorithmic based classification methods. We recognize that unsupervised clustering identifies transcriptionally unique populations, but the boundaries set by clustering do not always correspond with biologically relevant distinctions. As such, in our final annotations presented in **Table 2**, we collapsed some of the clusters into one cell category that we believed to be more biologically relevant. For example, five CD4^-^ monocyte populations were identified in our myeloid cell analysis, but we could not assign further identities to them, and in turn, annotated them all as one group of CD4^-^ monocytes. Furthermore, cell identification solely based on conical cell type markers (i.e., FOXP3 for Tregs and IFNG/TBX21 for Th1-like), proved ineffective in many instances. This is in part due to the low transcript abundance of transcription factors, cytokines, and chemokines in our dataset. Thus, many traditional gene markers for these cell types were not considered upregulated and were largely excluded from gene lists that define each cell population (as defined in **Table 3** and **Supplemental Data 1**/**2**). However, when subsetting and using pseudobulk approaches, we were able to better detect defining features, such as FOXP3 in regulatory T cells. This highlights the importance of using independent re-clustering on major cell populations and allowed us to provide detailed gene signatures for rare cell populations (available in **Supplemental Data 3**).

Despite our contributions, this study is not without limitations. For instance, granulocytes have been reported to be difficult to study using single-cell transcriptomics due to small transcriptomes, high RNase content, and sensitivity to sample processing (50). As a result, many research groups chose to filter out granulocyte populations. Despite this, we used single-cell RNA sequencing to characterize granulocyte populations and were able to identify distinct cell populations and report differentially expressed genes between healthy and OS dogs. While our data suggests there are biologically relevant differences between granulocyte populations in healthy and OS dogs, it is important to validate the findings reported here using molecular and functional assays. An additional limitation is the relatively low number of biological replicates and incomplete breed representation used to investigate disease induced changes in cell abundances. Future studies using alternative experimental approaches, such as flow cytometry, should be completed to validate conclusions presented here. Lastly, although we attempted to control for age between the healthy and OS dogs, we observed reductions in B cells and DN T cells which suggests the age difference between groups may have confounded our investigation of cancer associated changes.

We noted that our cell classifications are in discordance with those previously published for canine PBMCs (51). The only consistently annotated cell populations are B cells and platelets, while the remaining classifications differ between studies. For instance, the cluster classified as “monocytes” by Li et al. has high expression of CD3E which suggests a classification of T cells is more appropriate. Furthermore, the “T cell” cluster identified by Li et al. lacks CD3E expression, but has high S100A12/CD4 expression which likely corresponds to a neutrophil cluster (expression levels were determined using the web browser released by Li et al; http://120.79.46.200:81/Pandora/PBMC.html). The discrepancies between studies likely arose due to classification methods not taking into consideration the well-established phenomenon that, in dogs, neutrophils have high CD4 expression (34). Ultimately, cell classification should be completed using multiple approaches and biological insight when working with non-traditional animal models.

In summary, the data presented here serve two purposes. Firstly, the leukocyte subclassifications can act as a valuable resource for the scientific community to use in future research. For example, the proposed classifications provide detailed phenotypes of canine immune cells that can be used to inform the design of flow cytometry assays, bulk RNA sequencing deconvolution algorithms, and more (see Data and software availability section for details on use applications). Secondly, this study identified important differences in leukocyte abundances and transcriptomes in dogs with OS. Ultimately, our goal is that the database provided here will be used by researchers as a reference dataset as well as shed light on how cancer impacts circulating leukocytes.

## Data availability statement

Raw sequencing data (FASTQ format) is available on the NCBI Gene Expression Omnibus database under the accession number GSE225599. In addition to the raw data, processed Seurat objects are provided as supplemental files in the NCBI Gene Expression Omnibus database. The processed dataset is available for browsing at UCSC Cell Browser (https://canine-leukocyte-atlas.cells.ucsc.edu) (52). An associated GitHub page containing all the analysis code and software versions used to analyze the data presented in this article is available at (https://github.com/dyammons/Canine_Leukocyte_scRNA) (https://zenodo.org/record/7884518). Any additional data requests can be made by contacting the corresponding author or through the GitHub repository.

## Ethics statement

The animal study was reviewed and approved by Colorado State University (CSU) Institutional Animal Care and Use Committee and the CSU Clinical Review Board. Written informed consent was obtained from the owners for the participation of their animals in this study.

## Author contributions

Conception and design: DTA, SD. Experimentation and data acquisition: DTA, LSH, JK, KW. Data analysis and manuscript drafting: DTA, RAH, LSH, SD. Final approval of completed manuscript: All authors.

## Supplementary material

Supplementary Data Sheet 1 Complete gene list of transcriptomic signatures of all healthy immune cell types as determined using FindAllMarkers() with only.pos = TRUE and min.pct = 0.25.

Supplementary Data Sheet 2 Complete gene list of transcriptomic signatures of all cell types using the combined healthy-OS dataset, as determined using FindAllMarkers() with only.pos = TRUE, and min.pct = 0.25.

Supplementary Data Sheet 3 Results of all differential gene expression analysis presented in the manuscript in long data format. The column gs_base indicates the comparison (Y_VS_X) and the log2FoldChange column direction is positive for features up in Y and negative for those up in X.

Supplementary Data Sheet 4 Same contents as in **Table 3**, but in long data format for ease of use.
