# Supplementary material for: A single-cell RNA sequencing atlas of circulating leukocytes from healthy and osteosarcoma affected dogs
Source: Front Immunol. 2023 May 19;14:1162700. doi: 10.3389/fimmu.2023.1162700 (PMC10235626; doi:10.3389/fimmu.2023.1162700)
Supplement: Supplementary file 5 [file Presentation_1.pptx]

## Slide 1
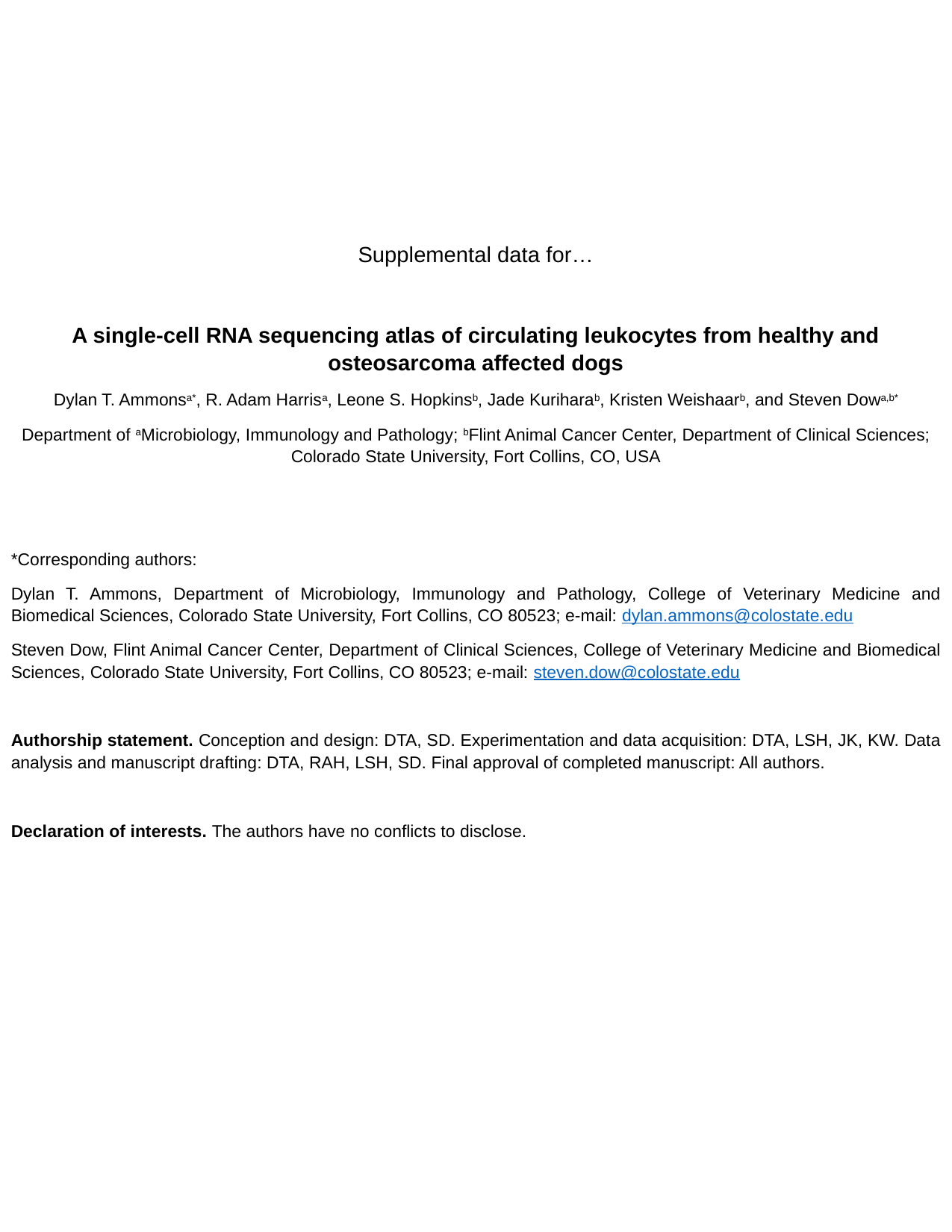

Supplemental data for…
A single-cell RNA sequencing atlas of circulating leukocytes from healthy and osteosarcoma affected dogs
Dylan T. Ammonsa*, R. Adam Harrisa, Leone S. Hopkinsb, Jade Kuriharab, Kristen Weishaarb, and Steven Dowa,b*
Department of aMicrobiology, Immunology and Pathology; bFlint Animal Cancer Center, Department of Clinical Sciences; Colorado State University, Fort Collins, CO, USA
*Corresponding authors:
Dylan T. Ammons, Department of Microbiology, Immunology and Pathology, College of Veterinary Medicine and Biomedical Sciences, Colorado State University, Fort Collins, CO 80523; e-mail: dylan.ammons@colostate.edu
Steven Dow, Flint Animal Cancer Center, Department of Clinical Sciences, College of Veterinary Medicine and Biomedical Sciences, Colorado State University, Fort Collins, CO 80523; e-mail: steven.dow@colostate.edu
Authorship statement. Conception and design: DTA, SD. Experimentation and data acquisition: DTA, LSH, JK, KW. Data analysis and manuscript drafting: DTA, RAH, LSH, SD. Final approval of completed manuscript: All authors.
Declaration of interests. The authors have no conflicts to disclose.

## Slide 2
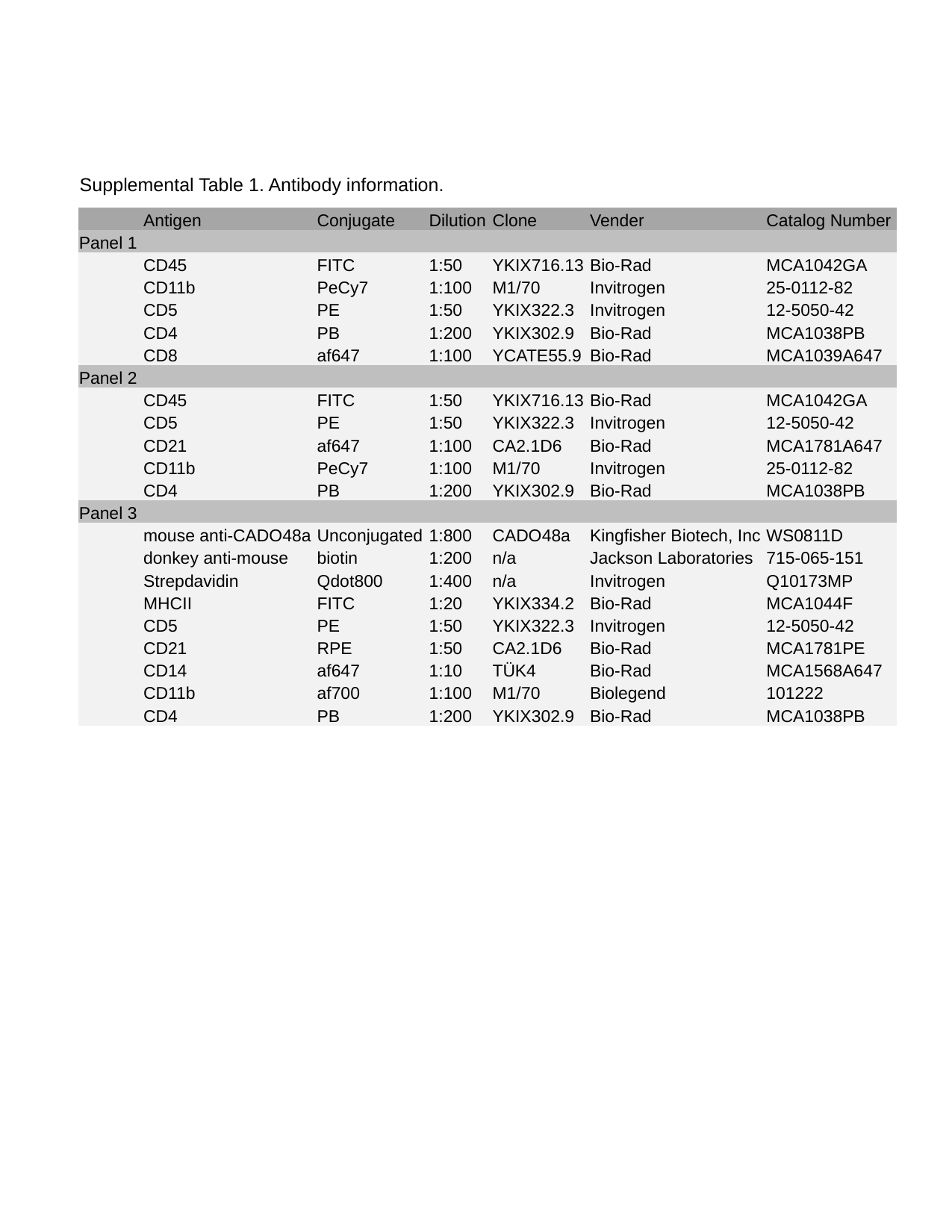

Supplemental Table 1. Antibody information.
| | Antigen | Conjugate | Dilution | Clone | Vender | Catalog Number |
| --- | --- | --- | --- | --- | --- | --- |
| Panel 1 | | | | | | |
| | CD45 | FITC | 1:50 | YKIX716.13 | Bio-Rad | MCA1042GA |
| | CD11b | PeCy7 | 1:100 | M1/70 | Invitrogen | 25-0112-82 |
| | CD5 | PE | 1:50 | YKIX322.3 | Invitrogen | 12-5050-42 |
| | CD4 | PB | 1:200 | YKIX302.9 | Bio-Rad | MCA1038PB |
| | CD8 | af647 | 1:100 | YCATE55.9 | Bio-Rad | MCA1039A647 |
| Panel 2 | | | | | | |
| | CD45 | FITC | 1:50 | YKIX716.13 | Bio-Rad | MCA1042GA |
| | CD5 | PE | 1:50 | YKIX322.3 | Invitrogen | 12-5050-42 |
| | CD21 | af647 | 1:100 | CA2.1D6 | Bio-Rad | MCA1781A647 |
| | CD11b | PeCy7 | 1:100 | M1/70 | Invitrogen | 25-0112-82 |
| | CD4 | PB | 1:200 | YKIX302.9 | Bio-Rad | MCA1038PB |
| Panel 3 | | | | | | |
| | mouse anti-CADO48a | Unconjugated | 1:800 | CADO48a | Kingfisher Biotech, Inc | WS0811D |
| | donkey anti-mouse | biotin | 1:200 | n/a | Jackson Laboratories | 715-065-151 |
| | Strepdavidin | Qdot800 | 1:400 | n/a | Invitrogen | Q10173MP |
| | MHCII | FITC | 1:20 | YKIX334.2 | Bio-Rad | MCA1044F |
| | CD5 | PE | 1:50 | YKIX322.3 | Invitrogen | 12-5050-42 |
| | CD21 | RPE | 1:50 | CA2.1D6 | Bio-Rad | MCA1781PE |
| | CD14 | af647 | 1:10 | TÜK4 | Bio-Rad | MCA1568A647 |
| | CD11b | af700 | 1:100 | M1/70 | Biolegend | 101222 |
| | CD4 | PB | 1:200 | YKIX302.9 | Bio-Rad | MCA1038PB |

## Slide 3
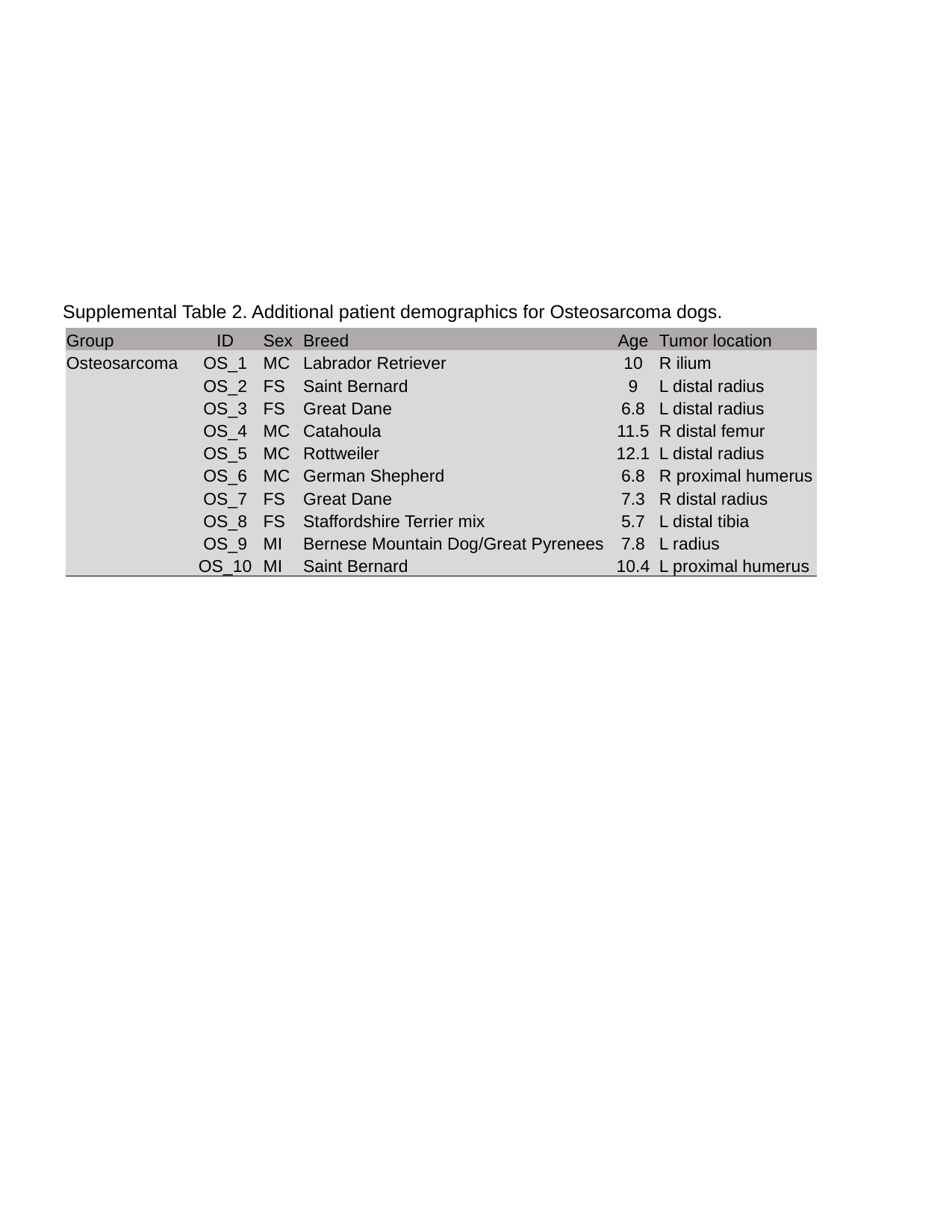

Supplemental Table 2. Additional patient demographics for Osteosarcoma dogs.
| Group | ID | Sex | Breed | Age | Tumor location |
| --- | --- | --- | --- | --- | --- |
| Osteosarcoma | OS\_1 | MC | Labrador Retriever | 10 | R ilium |
| | OS\_2 | FS | Saint Bernard | 9 | L distal radius |
| | OS\_3 | FS | Great Dane | 6.8 | L distal radius |
| | OS\_4 | MC | Catahoula | 11.5 | R distal femur |
| | OS\_5 | MC | Rottweiler | 12.1 | L distal radius |
| | OS\_6 | MC | German Shepherd | 6.8 | R proximal humerus |
| | OS\_7 | FS | Great Dane | 7.3 | R distal radius |
| | OS\_8 | FS | Staffordshire Terrier mix | 5.7 | L distal tibia |
| | OS\_9 | MI | Bernese Mountain Dog/Great Pyrenees | 7.8 | L radius |
| | OS\_10 | MI | Saint Bernard | 10.4 | L proximal humerus |

## Slide 4
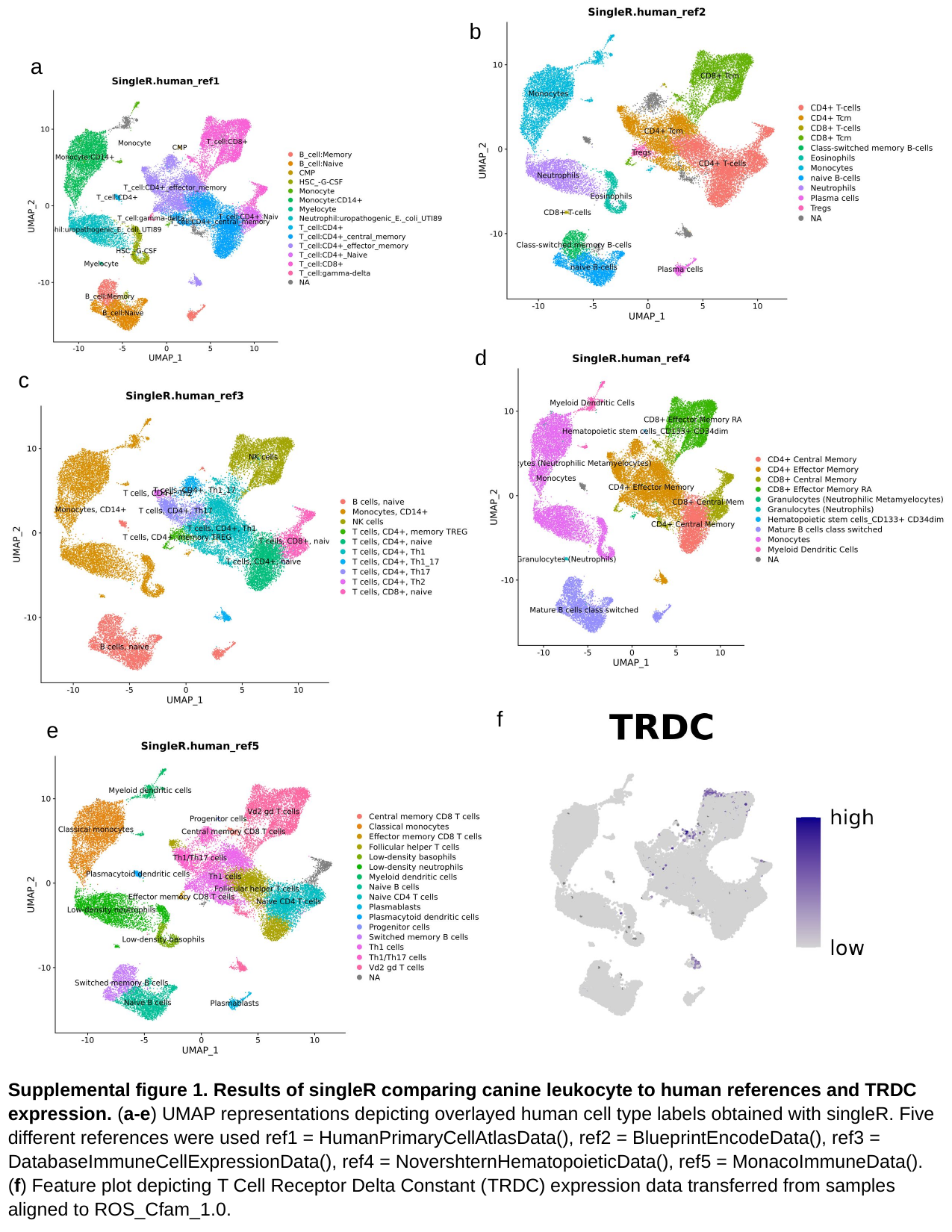

b
a
d
c
f
e
Supplemental figure 1. Results of singleR comparing canine leukocyte to human references and TRDC expression. (a-e) UMAP representations depicting overlayed human cell type labels obtained with singleR. Five different references were used ref1 = HumanPrimaryCellAtlasData(), ref2 = BlueprintEncodeData(), ref3 = DatabaseImmuneCellExpressionData(), ref4 = NovershternHematopoieticData(), ref5 = MonacoImmuneData(). (f) Feature plot depicting T Cell Receptor Delta Constant (TRDC) expression data transferred from samples aligned to ROS_Cfam_1.0.

## Slide 5
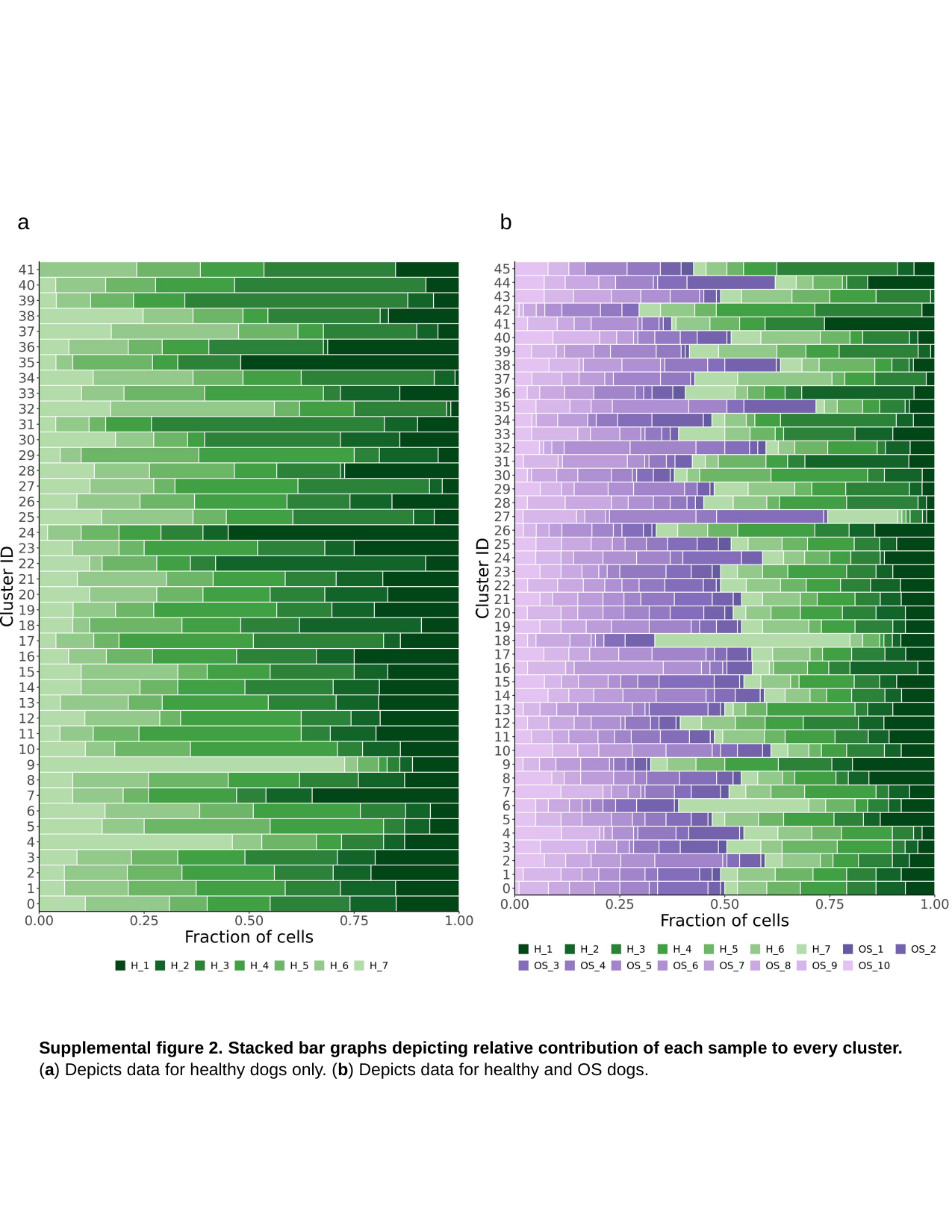

a
b
Supplemental figure 2. Stacked bar graphs depicting relative contribution of each sample to every cluster. (a) Depicts data for healthy dogs only. (b) Depicts data for healthy and OS dogs.

## Slide 6
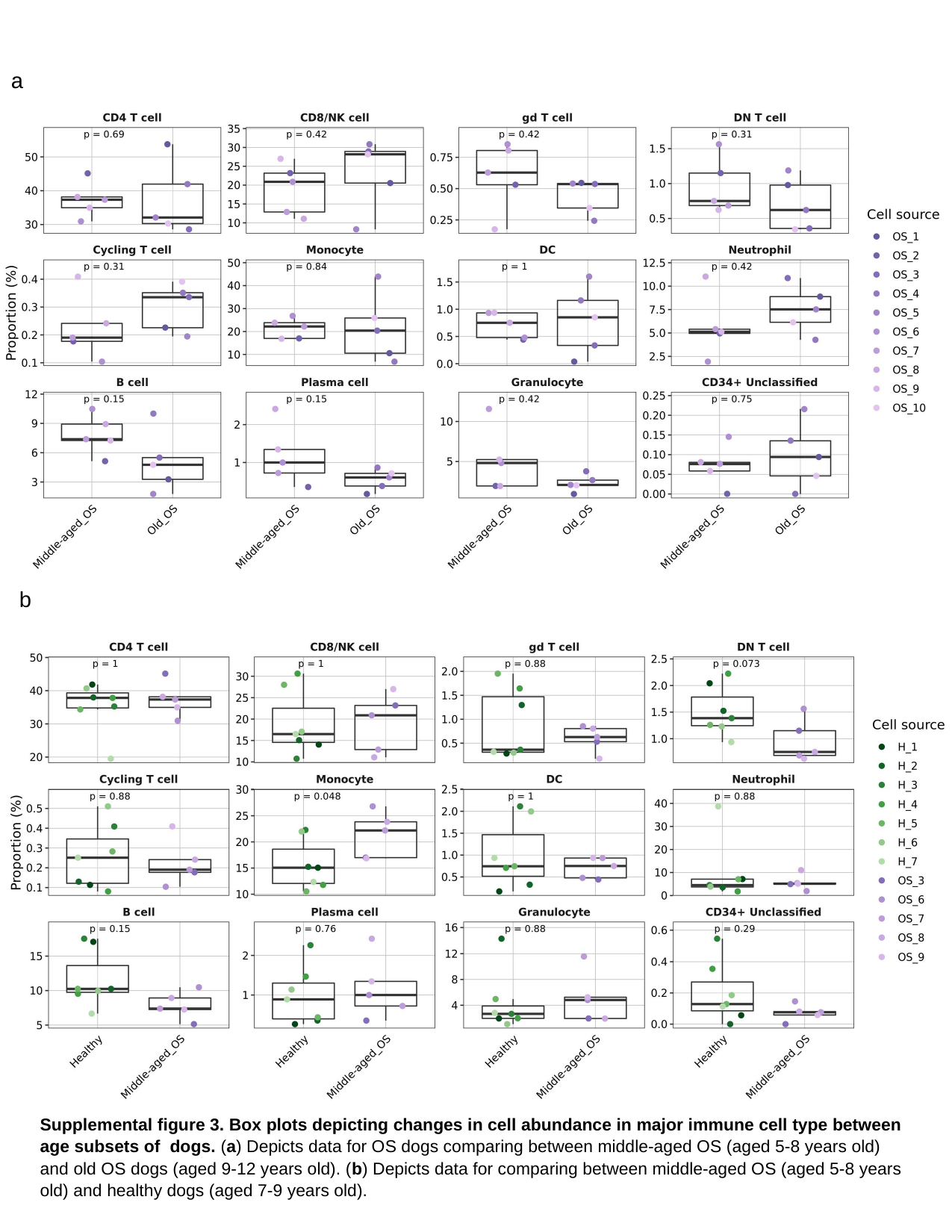

a
b
Supplemental figure 3. Box plots depicting changes in cell abundance in major immune cell type between age subsets of dogs. (a) Depicts data for OS dogs comparing between middle-aged OS (aged 5-8 years old) and old OS dogs (aged 9-12 years old). (b) Depicts data for comparing between middle-aged OS (aged 5-8 years old) and healthy dogs (aged 7-9 years old).

## Slide 7
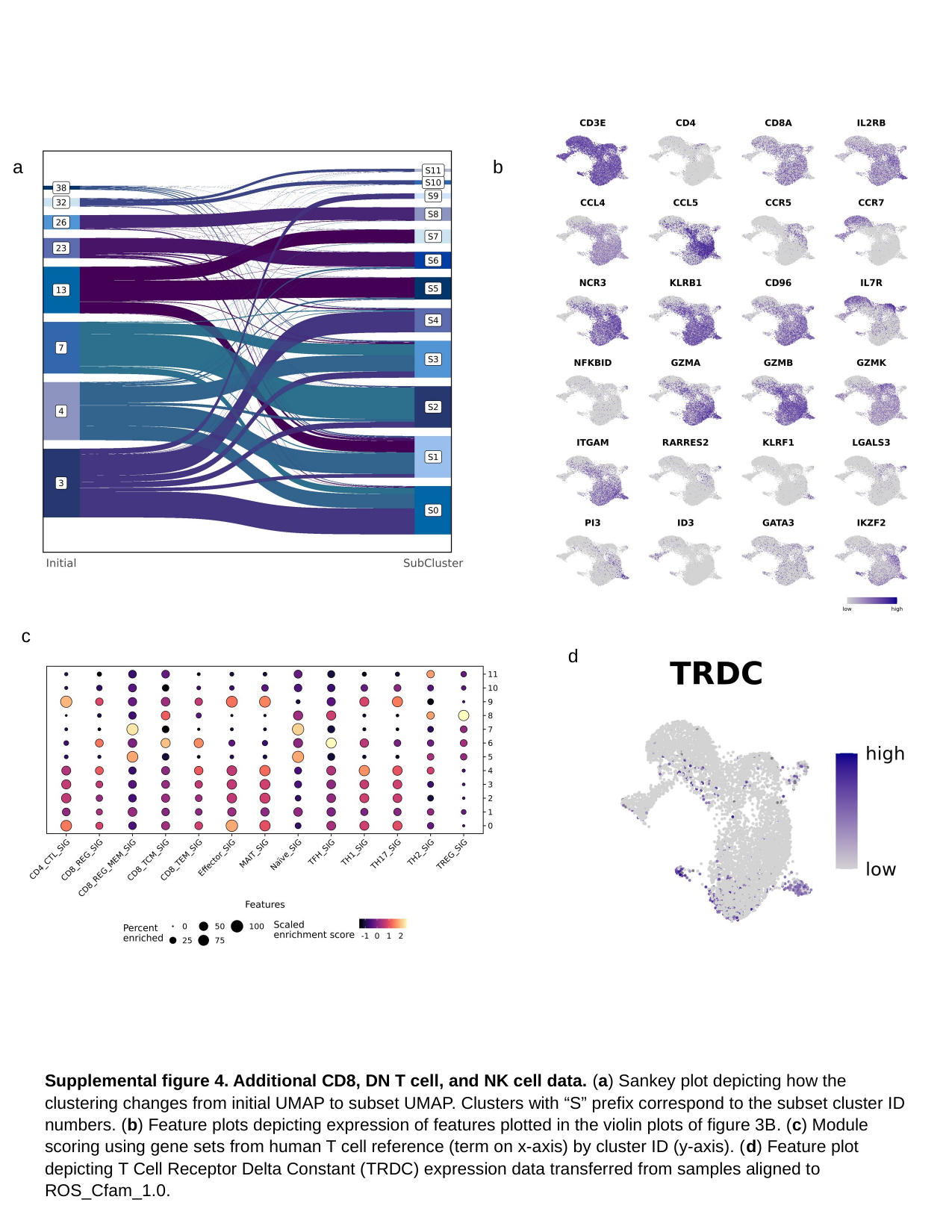

a
b
c
d
Supplemental figure 4. Additional CD8, DN T cell, and NK cell data. (a) Sankey plot depicting how the clustering changes from initial UMAP to subset UMAP. Clusters with “S” prefix correspond to the subset cluster ID numbers. (b) Feature plots depicting expression of features plotted in the violin plots of figure 3B. (c) Module scoring using gene sets from human T cell reference (term on x-axis) by cluster ID (y-axis). (d) Feature plot depicting T Cell Receptor Delta Constant (TRDC) expression data transferred from samples aligned to ROS_Cfam_1.0.

## Slide 8
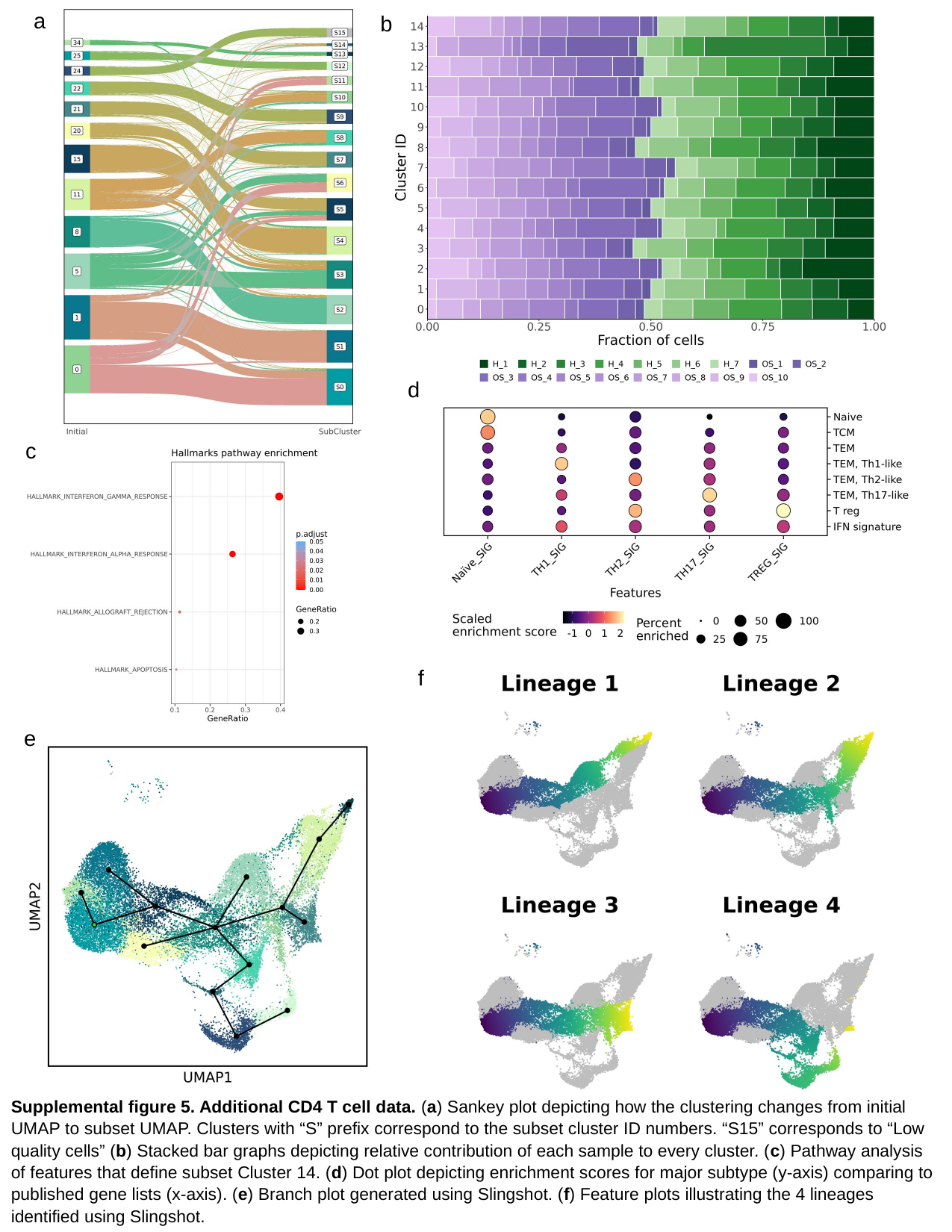

a
b
d
c
f
e
Supplemental figure 5. Additional CD4 T cell data. (a) Sankey plot depicting how the clustering changes from initial UMAP to subset UMAP. Clusters with “S” prefix correspond to the subset cluster ID numbers. “S15” corresponds to “Low quality cells” (b) Stacked bar graphs depicting relative contribution of each sample to every cluster. (c) Pathway analysis of features that define subset Cluster 14. (d) Dot plot depicting enrichment scores for major subtype (y-axis) comparing to published gene lists (x-axis). (e) Branch plot generated using Slingshot. (f) Feature plots illustrating the 4 lineages identified using Slingshot.

## Slide 9
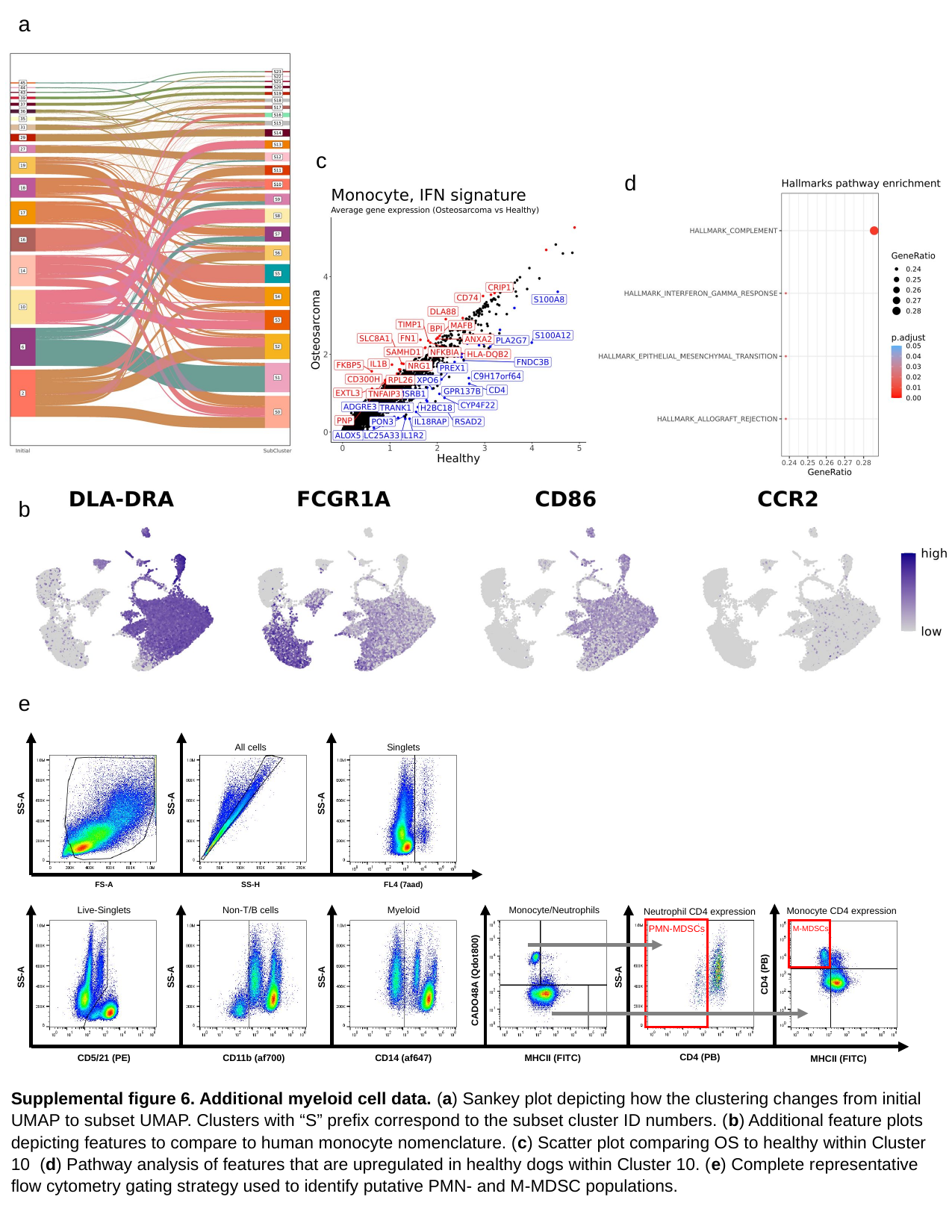

a
c
d
b
e
All cells
Singlets
SS-A
SS-A
SS-A
FS-A
SS-H
FL4 (7aad)
Live-Singlets
Non-T/B cells
Myeloid
Monocyte/Neutrophils
Monocyte CD4 expression
Neutrophil CD4 expression
PMN-MDSCs
M-MDSCs
CD4 (PB)
SS-A
SS-A
SS-A
SS-A
CADO48A (Qdot800)
CD4 (PB)
CD5/21 (PE)
CD11b (af700)
CD14 (af647)
MHCII (FITC)
MHCII (FITC)
Supplemental figure 6. Additional myeloid cell data. (a) Sankey plot depicting how the clustering changes from initial UMAP to subset UMAP. Clusters with “S” prefix correspond to the subset cluster ID numbers. (b) Additional feature plots depicting features to compare to human monocyte nomenclature. (c) Scatter plot comparing OS to healthy within Cluster 10 (d) Pathway analysis of features that are upregulated in healthy dogs within Cluster 10. (e) Complete representative flow cytometry gating strategy used to identify putative PMN- and M-MDSC populations.

## Slide 10
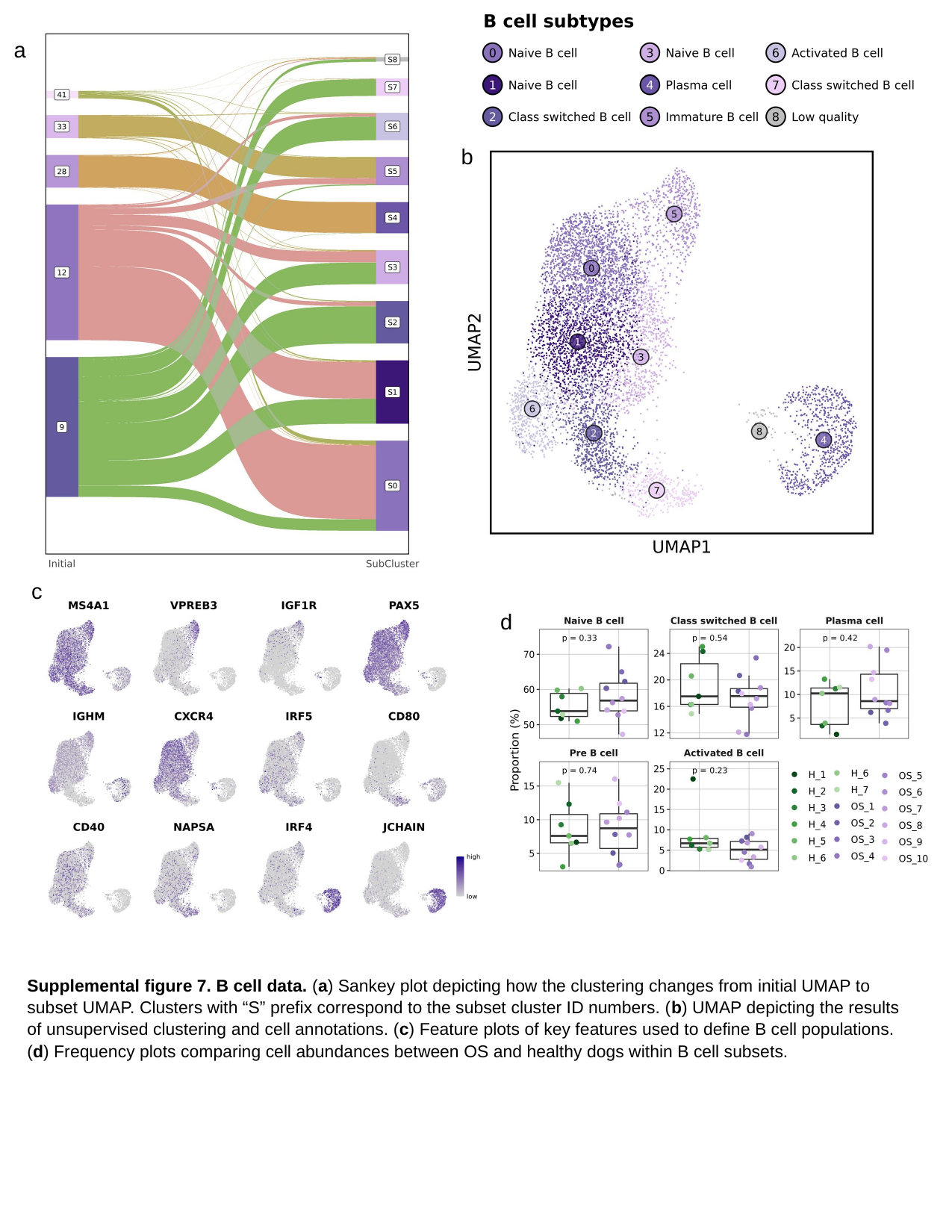

a
b
c
d
Supplemental figure 7. B cell data. (a) Sankey plot depicting how the clustering changes from initial UMAP to subset UMAP. Clusters with “S” prefix correspond to the subset cluster ID numbers. (b) UMAP depicting the results of unsupervised clustering and cell annotations. (c) Feature plots of key features used to define B cell populations. (d) Frequency plots comparing cell abundances between OS and healthy dogs within B cell subsets.

## Slide 11
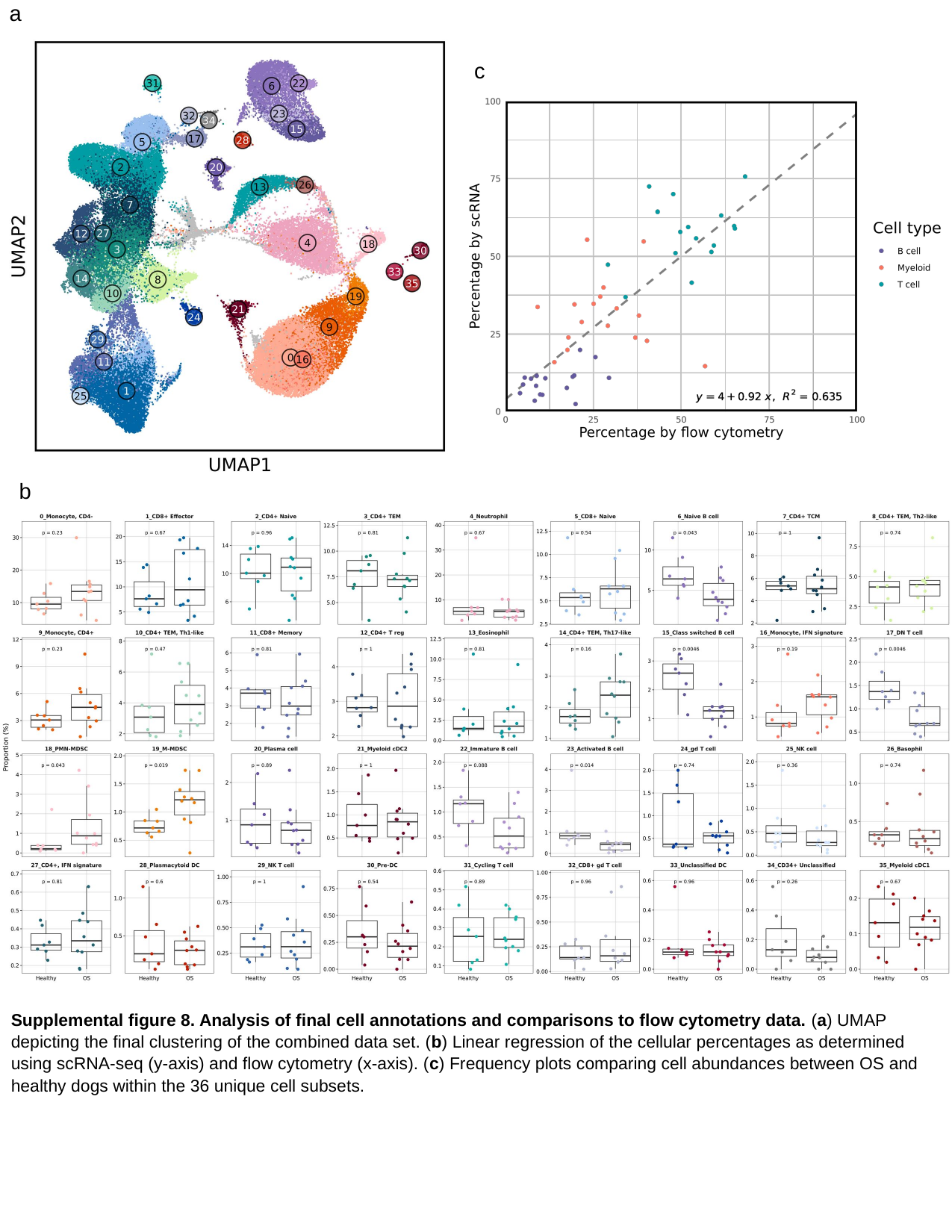

a
c
b
Supplemental figure 8. Analysis of final cell annotations and comparisons to flow cytometry data. (a) UMAP depicting the final clustering of the combined data set. (b) Linear regression of the cellular percentages as determined using scRNA-seq (y-axis) and flow cytometry (x-axis). (c) Frequency plots comparing cell abundances between OS and healthy dogs within the 36 unique cell subsets.

## Slide 12
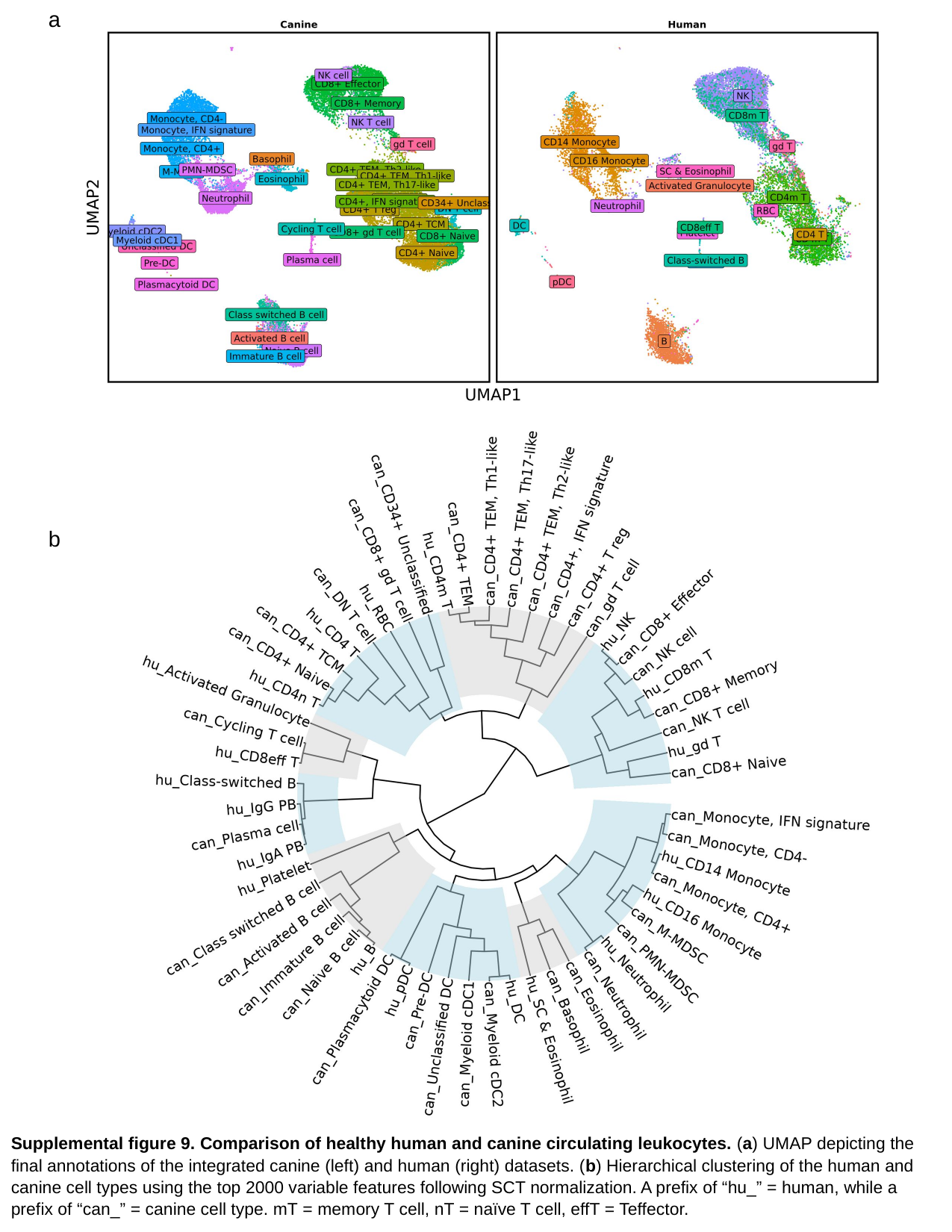

a
b
Supplemental figure 9. Comparison of healthy human and canine circulating leukocytes. (a) UMAP depicting the final annotations of the integrated canine (left) and human (right) datasets. (b) Hierarchical clustering of the human and canine cell types using the top 2000 variable features following SCT normalization. A prefix of “hu_” = human, while a prefix of “can_” = canine cell type. mT = memory T cell, nT = naïve T cell, effT = Teffector.
